# RsWRKY40 coordinates the cold stress response by integrating *RsSPS1*-mediated sucrose accumulation and the CBF-dependent pathway in radish (*Raphanus sativus* L.)

**DOI:** 10.1186/s43897-024-00135-x

**Published:** 2025-03-02

**Authors:** Sen Chen, Liang Xu, Yan Wang, Baozhen Mao, Xiaoli Zhang, Qiyu Song, Feng Cui, Yingbo Ma, Junhui Dong, Kai Wang, Hongyu Bi, Liwang Liu

**Affiliations:** 1 National Key Laboratory of Crop Genetics & Germplasm Enhancement and Utilization, Key Laboratory of Horticultural Crop Biology and Genetic Improvement (East China) of MOAR, College of Horticulture, Nanjing Agricultural University, Zhongshan Biological Breeding Laboratory, Nanjing, 210031 PR China; 2 College of Horticulture and Landscape Architecture, Yangzhou University, Yangzhou, 225009 PR China

**Keywords:** CBF, Cold stress response, Radish, RsWRKY40, Sucrose synthesis

## Abstract

Cold stress adversely affects crop growth and development. Radish is an important root vegetable crop, and its taproot formation is susceptible to low temperatures. However, the molecular basis of the cold stress response has not yet been fully dissected in radish. Here, a *sucrose phosphate synthase* gene (*RsSPS1*) was identified through a genome-wide association study and transcriptome analysis. *RsSPS1* was responsible for sucrose synthesis, and sucrose was shown to be involved in taproot growth, cambium activity, and cold tolerance in radish. *RsSPS1* regulated cambium activity and cold stress response by modulating sucrose content. Moreover, RsWRKY40 was identified as the upstream transcription activator of *RsSPS1* by binding to its promoter. RsWRKY40 functioned in cambium activity and cold tolerance by modulating *RsSPS1*-mediated sucrose accumulation. Furthermore, RsWRKY40 promoted the *RsCBF1* and *RsCBF2* expression levels, resulting in elevated cold resilience. RsWRKY40 also enhanced its own transcription, forming a positive auto-regulatory loop to regulate cold stress response in radish. Together, a transcription module of RsWRKY40 orchestrated cold stress response by integrating sucrose accumulation and the CBF-dependent pathway was uncovered. These findings would provide novel insight into the molecular mechanism underlying cold-responsive sucrose accumulation and cambium activity and facilitate the genetic improvement of cold tolerance in radish breeding programs.

**Supplementary Information:**

The online version contains supplementary material available at 10.1186/s43897-024-00135-x.

## Core

The radish taproot growth, cambium activity, and cold tolerance were promoted by exogenous sucrose. Knockdown of *RsSPS1* decreased SPS activity and sucrose content, which led to the attenuated PCNA signal, indicating that *RsSPS1* could regulate cambium activity and cold tolerance via sucrose synthesis in radish. RsWRKY40 positively regulated cold tolerance by modulation of *RsSPS1*-mediated sucrose synthesis and activation of *RsCBF1* and *RsCBF2* expression to coordinately regulate taproot cambium activity and cold stress response in radish.

## Gene and accession numbers

*RsWRKY40* (Rsa9g000650), *RsSPS1* (Rsa2g017240), *RsCBF1* (Rsa1g000520), *RsCBF2* (Rsa1g008490)

## Introduction

Cold stress is a globally challenging climate problem that adversely affects plant growth and development, geographical distribution, and crop productivity and quality (Lesk et al. 2016; Kidokoro et al. 2022). Cold stress leads to changes in secondary metabolites, photosynthesis, cell membrane integrity, and cell division activities, resulting in stunted plant growth and even entire plant death (Zhu 2016; Ding et al. 2019). The vascular cambium, a secondary meristem, is critical for radial root growth in cambium-driven root crops (Jang et al. 2015). The activity of vascular cambium is greatly plastic during the whole plant’s life. Cell division, expansion rates, cell-type specification, and differentiation of the cambium can be altered in response to environmental factors. These responses could protect the cambium against abiotic stresses including decreasing the impact of cold or drought stress via induction of stem cell quiescence during dry winter (Fischer et al. 2019). A range of conserved transcription factors, such as ERF-1, MYB15 and WRKY46, played critical roles in integration of environmental sensing and cambium cell division in root growth (Hoang et al. 2020). Root growth and development are inhibited under a low-temperature environment, resulting in a disordered cell structure, abnormal root structures, inactive meristem ability, and a decreased root biomass (Sun et al. 2017). Although the molecular mechanism underlying root growth and development has been extensively explored in *Arabidopsis thaliana* in response to cold stress (Karlova et al. 2021), little is known about the effect of low temperatures on cambium activity and root growth in radish and other cambium-driven root vegetable crops.

Sugar is involved in the regulation of root growth and development (Li et al. 2021) and is also correlated with freezing tolerance in plants (Bertrand et al. 2017). Sucrose, an important soluble sugar, is produced by photosynthesis in the shoot and acts as a signal molecule from shoots to roots for regulation of root growth (Kircher & Schopfer 2012, 2023). In addition, sucrose provides energy and metabolite platform to coordinate photosynthesis and root meristem activation and maintenance in plants (Xiong et al. 2013; Tong et al. 2022; Chen et al. 2022). The SPS genes are involved in sucrose synthesis and accumulation, regulating plants in response to various abiotic stress including cold and drought stress (Sami et al. 2016; Choudhary et al. 2022). For instance, in *Arabidopsis* *SPSA2* is involved in carbon partitioning and drought response via activation of the oxidative pentose phosphate pathway and accumulation of the total soluble sugar, respectively (Bagnato L et al. 2023). Soluble sugar is rapidly increased and used as a storage carbohydrate for respiratory energy when plants are exposed to low temperatures (Sami et al. 2016; Franzoni G et al. 2023). Soluble sugar accumulation depends on the import of sucrose from photosynthesizing leaves (Rolland et al. 2002). Sucrose phosphate synthase (SPS) (EC 2.4.1.14) is a key rate-limiting enzyme in sucrose synthesis and is positively correlated with cold tolerance in plants (Seydel et al. 2022). Increased SPS activity results in a higher capacity for sucrose synthesis, conferring freezing tolerance and improving photosynthesis under cold stress (Nägele et al. 2012). However, the cold-triggered transcriptional network of sucrose synthesis is largely unclear in root vegetable crops, including radish.

Plants are often affected by abiotic stress, including drought, cold, heat, and excess of salt or toxic metals in the soil. These unfavorable environmental conditions are major factors limiting crop productivity and threatening food security (Zhu 2016; Kidokoro et al. 2022). Plants have evolved a series of sophisticated and interconnected transcription regulatory networks to deal with cold stress (Ding et al. 2019; Kidokoro et al. 2022). The roles of the CBF transcription factor have been demonstrated to be required for cold acclimation (CA), whereby freezing tolerance is acquired after being exposed to nonfreezing temperatures in most plants (Guo et al. 2018; Zhang, et al. 2019a). Many transcription factors (TFs), including HY5, BZR1, and CAMTAs, have been identified as the upstream regulators of CBFs to regulate the cold stress response in plants (Kim et al. 2013; An et al. 2017; Li et al. 2017). In addition, CBF TFs are also involved in other abiotic stress response. CBF1, CBF2, CBF3 could directly interact with *GALS1* promoter to repress its expression, resulting in the elevated salt tolerance and decreased accumulation of β-1,4-Galactan (galactan), which could increase plant sensitivity to salt stress, synthesized by GALACTAN SYNTHASE1 (GALS1) (Yan et al. 2023). *MbCBF2* enhanced cold and salt tolerance by activating expression of gene that involved in cold signaling (*AtCOR15a*, *AtERD10*, *AtRD29a/b* and *AtCOR6.6/47*) and salt stress (*AtNCED3*, *AtCAT1*, *AtP5CS*, *AtPIF1/4* and *AtSnRK2.4*), respectively (Li et al. 2022). Moreover, plant-specific WRKY TFs are widely involved in the regulation of cold tolerance in plants. For instance, overexpressing *GmWRKY21* and *PmWRKY57* in *Arabidopsis* leads to enhanced cold tolerance (Zhou et al. 2008; Wang et al. 2023). *OsWRKY76*-overexpressing rice plants exhibit improved cold tolerance (Yokotani et al. 2013). WRKY transcription factors also regulated other abiotic stress response in plants (Jiang et al. 2017). LlWRKY22 played a positive role in regulation of thermotolerance by binding to *LlDREB2B* promoter to enhance its transcription level (Wu et al. 2022). Overexpression of *LlWRKY33* could improve heat tolerance in lily (Wu et al. 2023). *MbWRKY1* played a positive role in drought stress response via activation of oxidative enzymes and stress response gene in *Malus baccata* (L.) Borkh (Han et al. 2018b). MbWRKY4 could promote the expression of genes related to oxidative stress response (NtPOD, NtAPX and NtSOD) to enhance POD, APX and SOD activities under salt treatment (Han et al. 2018a). The overexpression of *MxWRKY55* enhanced the salt and Fe tolerance in *M. xiaojinensis* (Han et al. 2020). Overexpression of *MxWRKY53* could promote salt and Fe tolerance in *Arabidopsis* (Han et al. 2022). VhWRKY44 positively regulated salt and cold stress response via increasing SOD, POD and CAT activities and proline content in grape (Zhang et al. 2024). In addition to regulating plant responses to abiotic stress, WRKY TFs were also identified to modulate sugar accumulation in plants. PuWRKY31 binds to the *PuSWEET15* promoter to activate its transcription, thus regulating sucrose levels in pear fruit (Li et al. 2020b). Nevertheless, how WRKY TFs orchestrate cold-induced sucrose accumulation and CBF signaling for the cold stress response is largely unknown in cambium-driven root vegetable crops.

Radish (*Raphanus sativus* L.) is an important root vegetable crop worldwide. Its taproot is used as an edible storage organ that contains abundant vitamins, mineral elements, and carbohydrates. Taproot formation is determined by the constitutively undifferentiated division cells in the vascular cambium, which generate daughter cells in periclinal directions that become the xylem and phloem of radish taproots. From winter to early spring, the low temperature is a serious limiting factor inhibiting cambial cell division, which results in an inactive vascular cambium and stunted root growth. To explore the transcription regulatory mechanism underlying the cambium activity and cold tolerance in radish, the molecular regulation of *RsSPS1*-modulated sucrose synthesis and cambium activity was first investigated in this study. RsWRKY40 has been identified as the upstream transcriptional activator of *RsSPS1*, and it regulates the cambium activity and cold tolerance by modulating the sucrose content. Moreover, RsWRKY40 positively regulates cold tolerance by activating *RsCBF1* and *RsCBF2* expression in radish. These findings provide insight into the molecular transcription regulatory underlying sucrose accumulation, which confers enhanced cambium activity and cold tolerance, and will facilitate the genetic development of elite cultivars with cold resilience in radish and other root vegetable crops.

## Results

### *RsSPS1* is a candidate gene associated with the sucrose content

**Fig. 1:**
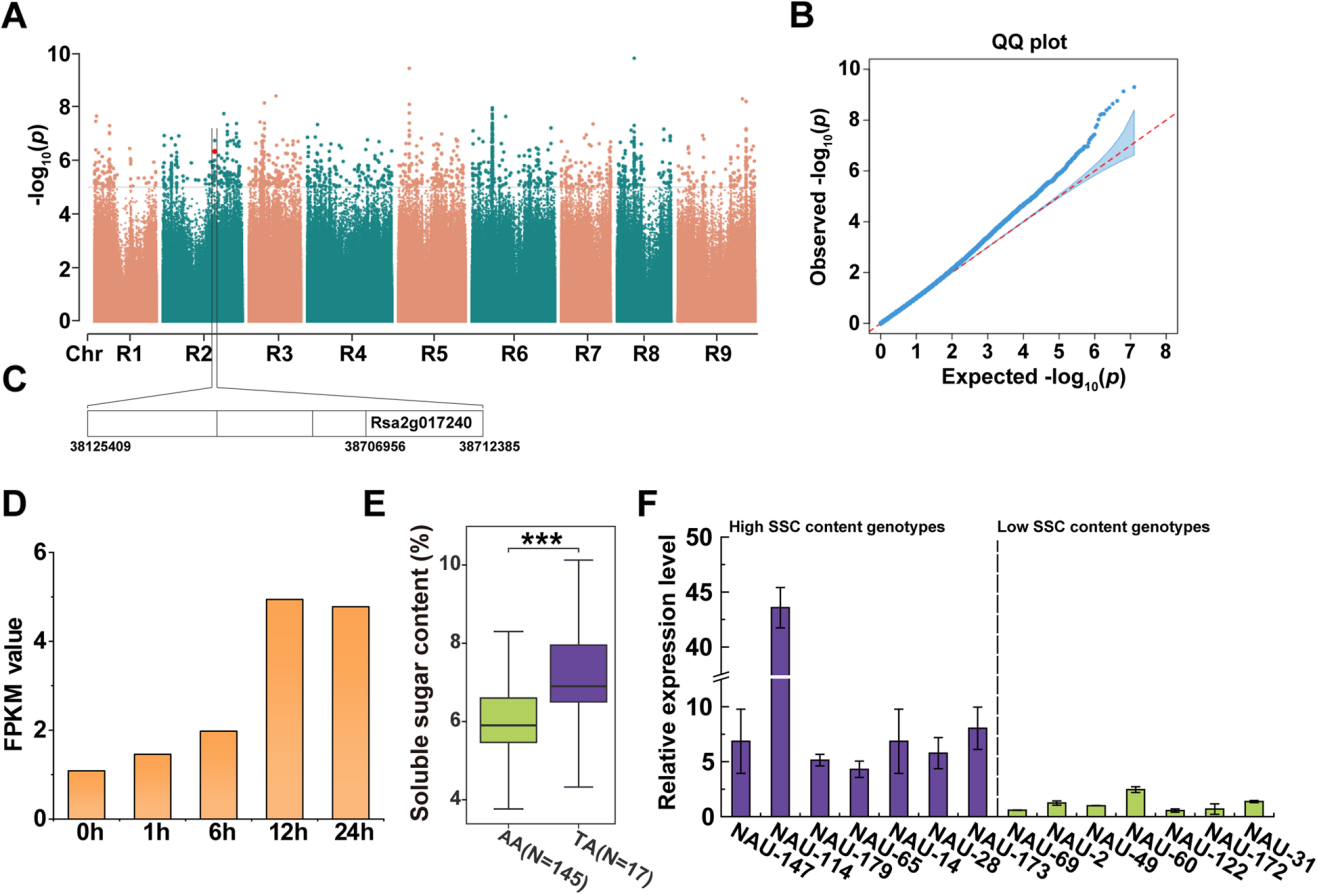
A genome-wide association study (GWAS) for the soluble sugar content in radish. **A**, **B** Results of GWAS for the soluble sugar content exhibited by the Manhattan plots (**A**) and quantile–quantile plots (**B**). **C ***RsSPS1* (Rsa2g017240) was mapped to a physical position on chromosome 2. **D** The *RsSPS1* expression pattern in the RNA-seq analysis during different cold treatments. The *RsSPS1* expression level was indicated by FPKM values (fragments per kilobase of exon model per million mapped fragments). **E** The soluble sugar content of radish accessions grouped by the lead SNP. The significant differences between two groups were determined by Student’s *t*-test (***, *P* < 0.001), N indicates the number of accessions with the same genotype (N = 145 for genotype AA; N = 17 for genotype TA). **F** Expression levels of *RsSPS1* in high SSC content (TA) and low SSC content (AA) radish genotypes, detected by RT-qPCR. Data are presented as the mean ± SD; *n* = 3

The phenotype data of the soluble sugar content (SSC) related to the sucrose content showed a continuous distribution (Fig. S1). GWAS results showed that a lead SNP was detected on chromosome 2 (Chr2: 38,125,409 nt), and significantly associated with one gene *Rsa2g017240* which was closely related to the phenotype (Fig. 1A–C). The SSC content of radish accessions carrying the sequence TA showed significantly higher level than those with the sequence AA (Fig. 1E). Totally seven individuals with two genotypes of TA and AA, respectively, were selected for gene expression analysis. *RsSPS1* exhibited higher expression level in high SSC content genotypes carrying the TA sequence than in low SSC content genotypes with AA sequence, respectively (Fig. 1F). The CDS of *Rsa2g017240* was 3144 bp in length and encoded a sucrose-phosphate synthase. Its amino acid sequence displayed the highest similarity with AtSPS1F of *A*. *thaliana* (Fig. S2). Therefore, *Rsa2g017240* was named *RsSPS1*.

For dissection of the molecular mechanism underlying radish in response to cold stress, the transcript level of *RsSPS1* was assessed by using transcriptome data. The expression levels of *RsSPS1* were highly increased among cold treatments, as indicated using RNA sequencing (RNA-seq) analysis (Fig. 1D). This was consistent with the RT-qPCR results in which the transcript level of *RsSPS1* was notably upregulated with the extension of cold treatment (Fig. S3A), indicating that *RsSPS1* might play a critical role in sucrose synthesis under low temperatures in radish.

### Sucrose is critical for taproot growth and cold tolerance in radish

**Fig. 2:**
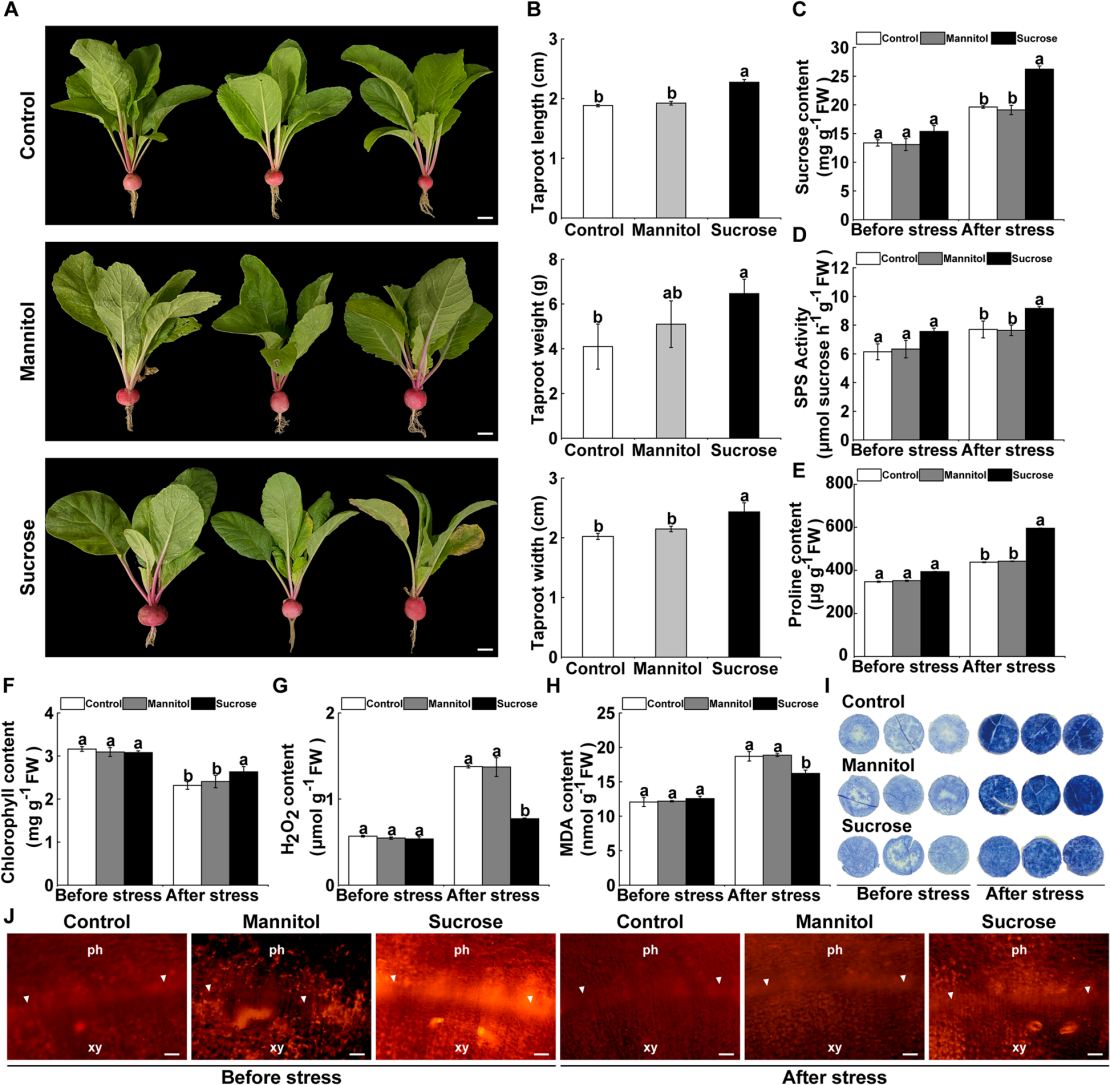
Sucrose is involved in taproot growth and cold tolerance in radish. **A**, **B** The morphology (**A**) and taproot length, weight and width (**B**) between radish supplied with exogenous 60 mM sucrose solution and the control plants treated with H_2_O and mannitol. Bar: 2 cm. **C**, **D** The levels of sucrose content (**C**) and sucrose phosphate synthase (SPS) activity (**D**) in the control and sucrose-supplied radish plants. **E**-**I** The content of proline (**E**), chlorophyll (**F**), H_2_O_2_ (**G**), malondialdehyde (MDA) (**H**) and in situ histochemical staining of nitro blue tetrazolium (NBT) (**I**) in the controls and sucrose-treated radish before and after cold stress. **J** PCNA immunolocalization in the vascular cambium cells in the taproots of the lines examined before and after cold treatment. ph, phloem; xy, xylem; arrowhead, cambium. Bar: 200 μm. For B-H, data represented the mean ± SD; *n* = 3

To explore the role of sucrose in radish taproot growth, a 60 mM sucrose solution was applied to radish plants. The taproot length, weight, and width increased in radish treated with exogenous sucrose compared with the controls (Fig. 2A, B). The sucrose content and SPS activity increased in sucrose-supplied radish compared to the controls (Fig. 2C, D). A higher proline and chlorophyll content, accompanied by a lower reactive oxygen species (ROS) and MDA content, were observed in the sucrose-supplied radish after cold stress (Fig. 2E–I).

Active cell division in the vascular cambium is critical for cambium-driven radish taproot formation. To explore the role of sucrose in cambial cell proliferation activities, an immunolocalization assay was performed using an antibody against PCNA, which is extensively involved in DNA replication and whose abundance is highly enriched in actively dividing cells. An increased PCNA signal in the cambium cells was detected in sucrose-treated taproots compared with the controls with or without cold stress (Fig. 2J). These results indicate that sucrose is involved in radish taproot growth, cold tolerance regulation, and cell division activity maintenance in the vascular cambium.

### *RsSPS1* positively regulates cold stress response in radish

**Fig. 3:**
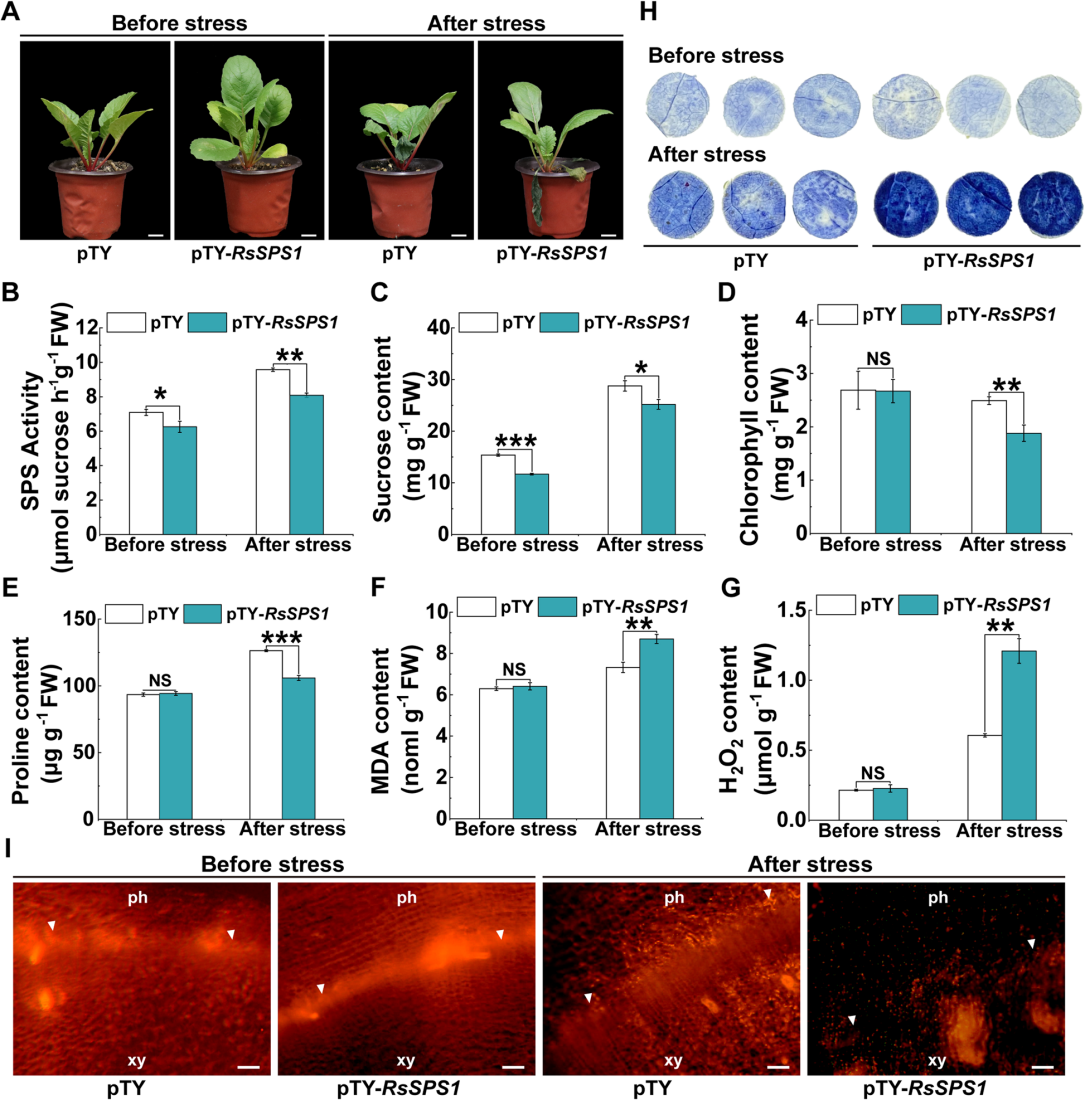
Silencing of *RsSPS1* attenuates the cold tolerance in radish. **A**-**C** The phenotype (**A**), SPS activity (**B**) and sucrose content (**C**) in the pTY empty vector (control) plant and pTY-*RsSPS1* silenced radish lines before and after cold stress. Bar: 2 cm. **D**-**F** The content of chlorophyll (**D**), proline (**E**), and malondialdehyde (MDA) (**F**) in the control and VIGS lines before and after cold treatment. **G**, **H** The H_2_O_2_ content (**G**) and in situ histochemical staining of nitro blue tetrazolium (NBT) (**H**) in the experimental lines with or without cold stress. **I** Detection of PCNA immunolocalization in the cambium cells in the control and *RsSPS1*-silenced radish taproots. ph, phloem; xy, xylem; arrowhead, cambium. Bar: 200 μm. Asterisks indicate the significant differences between the VIGS and control plants (*P < 0.05, **P < 0.01, ***P < 0.001). Data are presented as the mean ± SD; *n* = 3

To verify the function of *RsSPS1* in the regulation of cold tolerance, it was knocked down in radish plants using VIGS. The transcript level of *RsSPS1* decreased in *RsSPS1*-silenced radish (Fig. S4). The expression levels of *RsSPS1* homologs, including *Rsa7g012830*, *Rsa4g016410*, *Rsa3g027520*, and *Rsa1g002860*, were not noticeably changed in the *RsSPS1-*VIGS lines, suggesting that *RsSPS1* was specifically silenced in the VIGS plants (Fig. S4). Silencing of *RsSPS1* significantly impaired SPS activity and reduced the sucrose content compared with the controls (Fig. 3B, C). Transient overexpression of *RsSPS1* in radish resulted in an elevated SPS activity and sucrose content (Fig. S5A, B), indicating that *RsSPS1* is associated with sucrose content. The elevated proline content and reduced MDA and ROS content were also measured in *RsSPS1*-overexpressing plants in comparison with the controls after cold stress (Fig. S5). *RsSPS1*-silenced plants showed leaf wilting and curling relative to the pTY control plants after cold stress (Fig. 3A). Lower chlorophyll and proline content, higher MDA and H_2_O_2_ content, and in situ O_2_^∙−^ accumulation were observed in the knockdown lines compared to the controls after cold stress (Fig. 3D–H), suggesting that *RsSPS1* positively regulated cold tolerance in radish. Moreover, a decreased PCNA signal was detected in *RsSPS1*-silenced radish taproot compared to the controls after cold treatment (Fig. 3I), indicating that *RsSPS1* played a positive role in cambial cell division activity in response to cold stress.

### Identification of the upstream transcription factor of *RsSPS1*

**Fig. 4:**
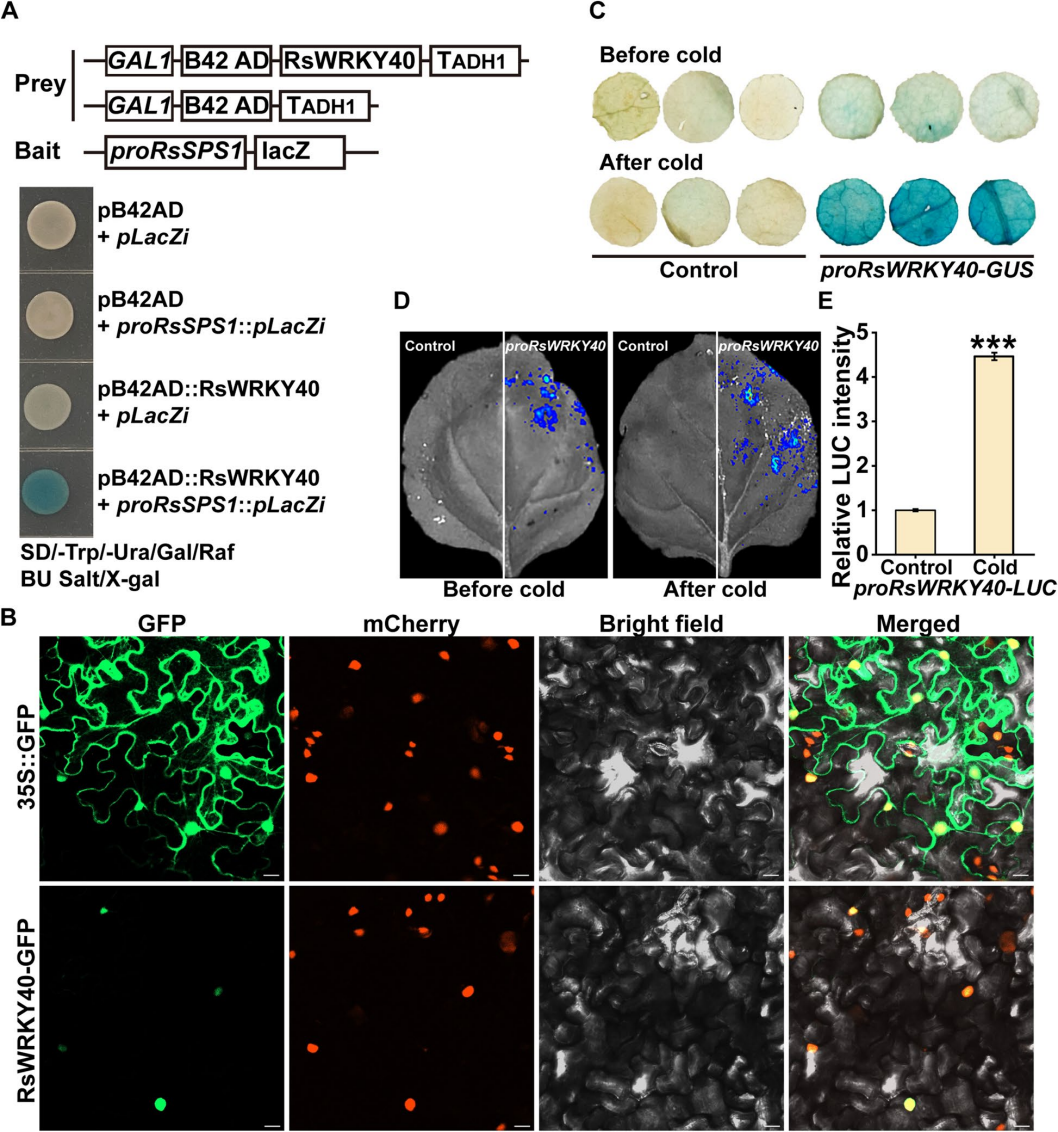
RsWRKY40 is a cold-induced nuclear transcription factor. **A** A Yeast one-hybrid (Y1H) assay used for the determination of the interaction between the bait and the prey. The schematic diagram of bait and prey constructs (upper panel). RsWRKY40 binds to the *RsSPS1* promoter (lower panel). Yeast cells co-transformed with the prey and bait constructs were grown on the SD –Ura/–Trp medium containing X-gal (5-Bromo-4-chloro-3-indolyl β-D-galactopyranoside). **B** The subcellular localization of RsWRKY40. The *Nicotiana benthamiana* epidermal cells from the leaves were transformed with 35S::GFP or 35S::RsWRKY40-GFP plasmids. The 35S::GFP infusion protein and 35S::H2B-mCherry marker were used as the negative control and confirmation of the nucleus location, respectively. Bar: 20 μm. GFP, green fluorescent protein. **C** The analysis of GUS staining in *N. benthamiana* leaves expressing *proRsWRKY40::GUS* vector before and after cold treatment. **D** Promoter activity of *RsWRKY40* was examined by firefly luciferase (LUC) reporter assay in *N. benthamiana* leaves with and without cold treatment. **E ***RsWRKY40* promoter activity was elevated by cold treatment. The analysis of relative LUC intensity was performed by using the software of Clinx IVScopeEQ Capture. The relative values were obtained by comparing the leaves between stress and no stress. Data represented as the mean ± SD; *n* = 3 (***P < 0.001, Student’s *t*-test)

**Fig. 5:**
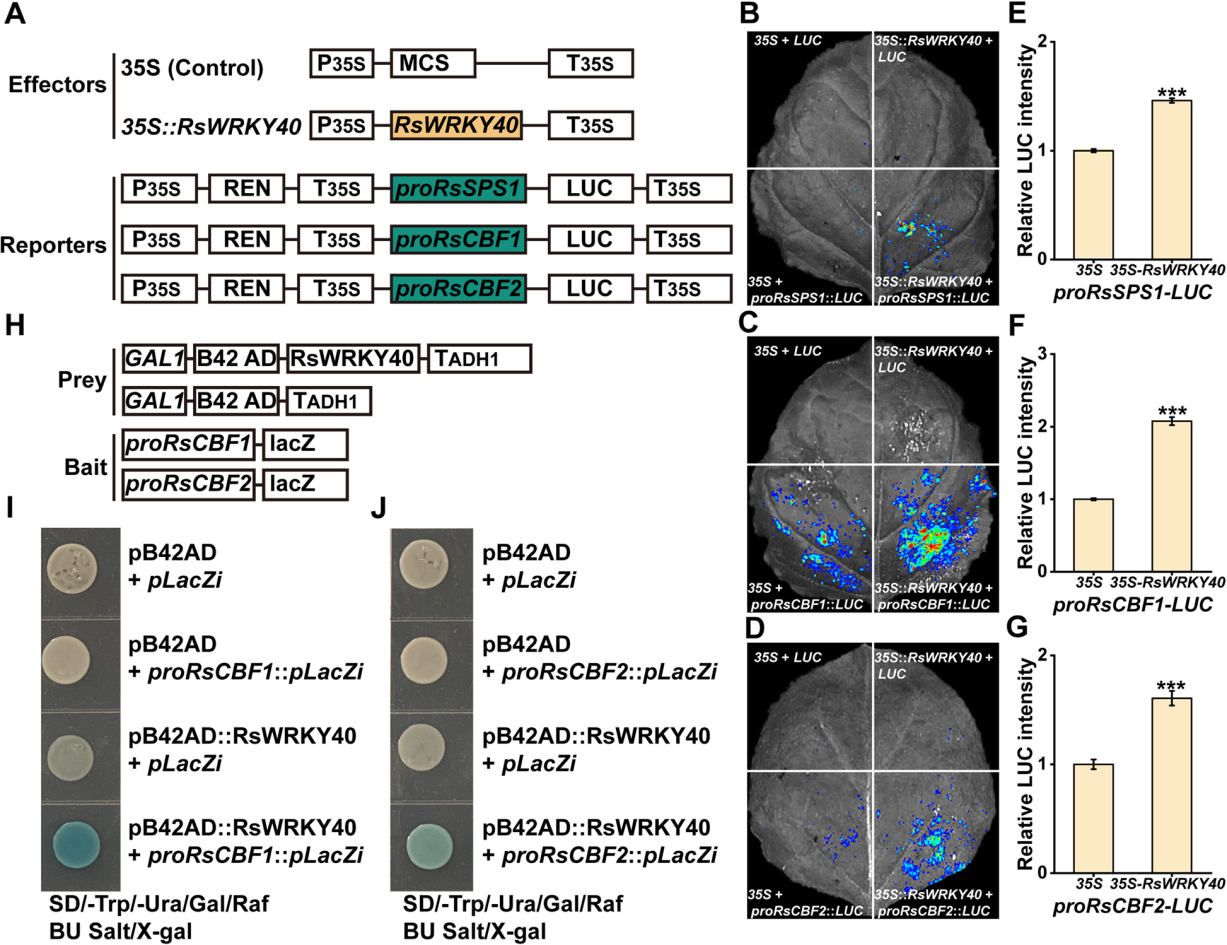
RsWRKY40 binds to the promoter of *RsSPS1*, *RsCBF1* and *RsCBF2* to activate their expression. **A** The schematic diagram of the effector and reporter constructs used in the dual luciferase assay. P35S and T35S, the CaMV *35S* promoter and terminator, respectively. LUC, firefly luciferase. REN, Renilla luciferase. MCS, multiple cloning sites. **B**-**D** Promoter activation of *RsSPS1* (**B**), *RsCBF1* (**C**) and *RsCBF2* (**D**) as evidenced by LUC images. The detection of the LUC signal was performed in *N. benthamiana* leaves co-expressed effectors and reporters after 48 h post-infiltration. **E**–**G** The activation of *RsSPS1-LUC* (**E**), *RsCBF1-LUC* (**F**) and *RsCBF2-LUC* (**G**) reporter by *35S*::*RsWRKY40* effector in luciferase assays. Data are presented as the mean ± SD; *n* = 3 (***P < 0.001, Student’s *t*-test). **H** The schematic diagram of the prey and bait constructs used in the Y1H assay. *GAL1*, GAL1 promoter. B42 AD, B42 transcriptional activator. TADH1, ADH1 terminator. lacZ, *E. coli* lacZ gene encoding *β*-galactosidase. **I**, **J** RsWRKY40 binds to the *RsCBF1* (**I**) and *RsCBF2* (**J**) promoter. The yeast one-hybrid analysis, using pB42AD-RsWRKY40 as the prey vector, *proRsCBF1-lacZ* and *proRsCBF2-lacZ* as the bait vectors

To identify the upstream transcription regulator of *RsSPS1* for the cold stress response, the Y1H assay was performed using the *RsSPS1* promoter as bait. Several W-box elements were identified in the *RsSPS1* promoter (Fig. S10D). Many WRKY TFs played pivotal roles in regulating cold stress response in plants via modulating the gene expression by binding to the W-box elements on their targeted gene promoter. We therefore speculated that *RsSPS1* might be regulated by WRKY TFs. An interaction was identified between the WRKY transcription factor (Rsa9g000650, RsWRKY40) and the *RsSPS1* promoter (Figs. 4A and S6A). The conserved WRKYGQK domain and a C_2_H_2_ finger motif were found within the RsWRKY40 protein sequence (Fig. S6B, C). Subsequently, a luciferase reporter assay was performed to further determine whether RsWRKY40 could activate or suppress *RsSPS1* expression. The LUC signal was significantly elevated by co-expression of the effector (*35S::RsWRKY40*) and the reporter (*proRsSPS1-LUC*) compared to the controls (Fig. 5A, B, E), indicating that RsWRKY40 directly activates *RsSPS1* expression by binding to its promoter.

Interestingly, the GFP fluorescence signal from *N*. *benthamiana* leaves infiltrated with the empty vector was observed throughout the cells, whereas GFP fluorescence from the RsWRKY40-GFP protein was exclusively detected in the nucleus, indicating that RsWRKY40 is located in the nucleus (Fig. 4B). To examine the *RsWRKY40* promoter activity, the *proRsWRKY40*-*GUS* fusion plasmid was transiently expressed in* N*. *benthamiana* leaves. The GUS activities in leaves expressing the *proRsWRKY40*-*GUS* plasmid were elevated after cold treatment (Fig. 4C). Furthermore, the LUC intensity in the *proRsWRKY40-LUC* fusion constructs was elevated by cold treatment (Fig. 4D, E), indicating that the *RsWRKY40* promoter could be activated by low temperatures. Therefore, RsWRKY40 is a cold-induced nuclear protein.

### RsWRKY40 modulates *RsSPS1*-mediated sucrose synthesis in response to cold stress

**Fig. 6:**
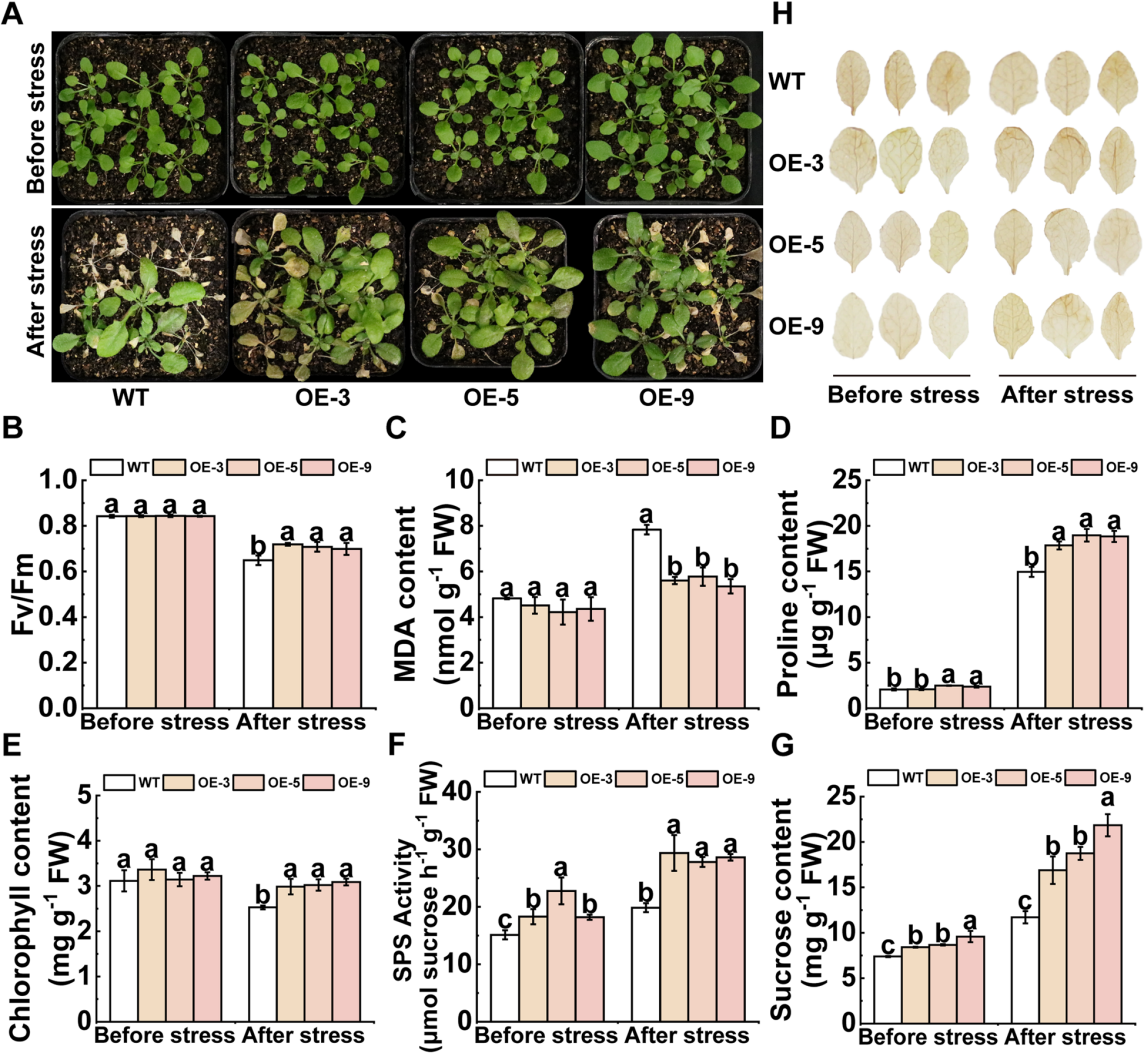
Overexpression of *RsWRKY40* confers enhanced cold tolerance in transgenic plants. **A** Phenotype of *RsWRKY40*-overexpressing transgenic *Arabidopsis* plants and wild type with or without freezing treatment and subsequent recovery. **B**-**E** The *Fv/Fm* ratios (**B**), malondialdehyde (MDA) (**C**), proline content (**D**), chlorophyll content (**E**) of WT and transgenic plants overexpressing *RsWRKY40* measured before and after cold stress. **F**, **G** The SPS activity (**F**) and sucrose content (**G**) in WT and *RsWRKY40*-overexpressing transgenic plants before and after cold stress. **H** In situ 3,3'-Diaminobenzidine (DAB) staining of the representative leaves collected from the WT and transgenic lines before and after cold treatment. Data represented the mean ± SD; *n* = 3

The *RsWRKY40* expression level was steadily elevated when exposed to cold stress, suggesting that RsWRKY40 is a cold-responsive transcription factor (Fig. S3B). Transgenic *Arabidopsis* plants overexpressing *RsWRKY40* (*RsWRKY40*-OE) were used to explore the role of *RsWRKY40* in the regulation of cold tolerance. Although the transgenic plants were hardly distinguishable from the wild type (WT) under normal conditions, the *RsWRKY40*-OE lines showed less leaf wilting than the WT lines after cold stress, and the transgenic plants exhibited considerably lower MDA accumulation and a higher chlorophyll and proline content as well as improved *Fv*/*Fm* ratios compared to the controls after cold stress (Fig. 6A–E). The SPS activity and sucrose content in transgenic plants were higher than those in controls before and after cold treatment (Fig. 6F, G). These results agree with those of transient *RsWRKY40* overexpression in radish plants (Fig. S7), indicating that *RsWRKY40* is critical for modulating cold tolerance and promoting sucrose accumulation in radish.

**Fig. 7:**
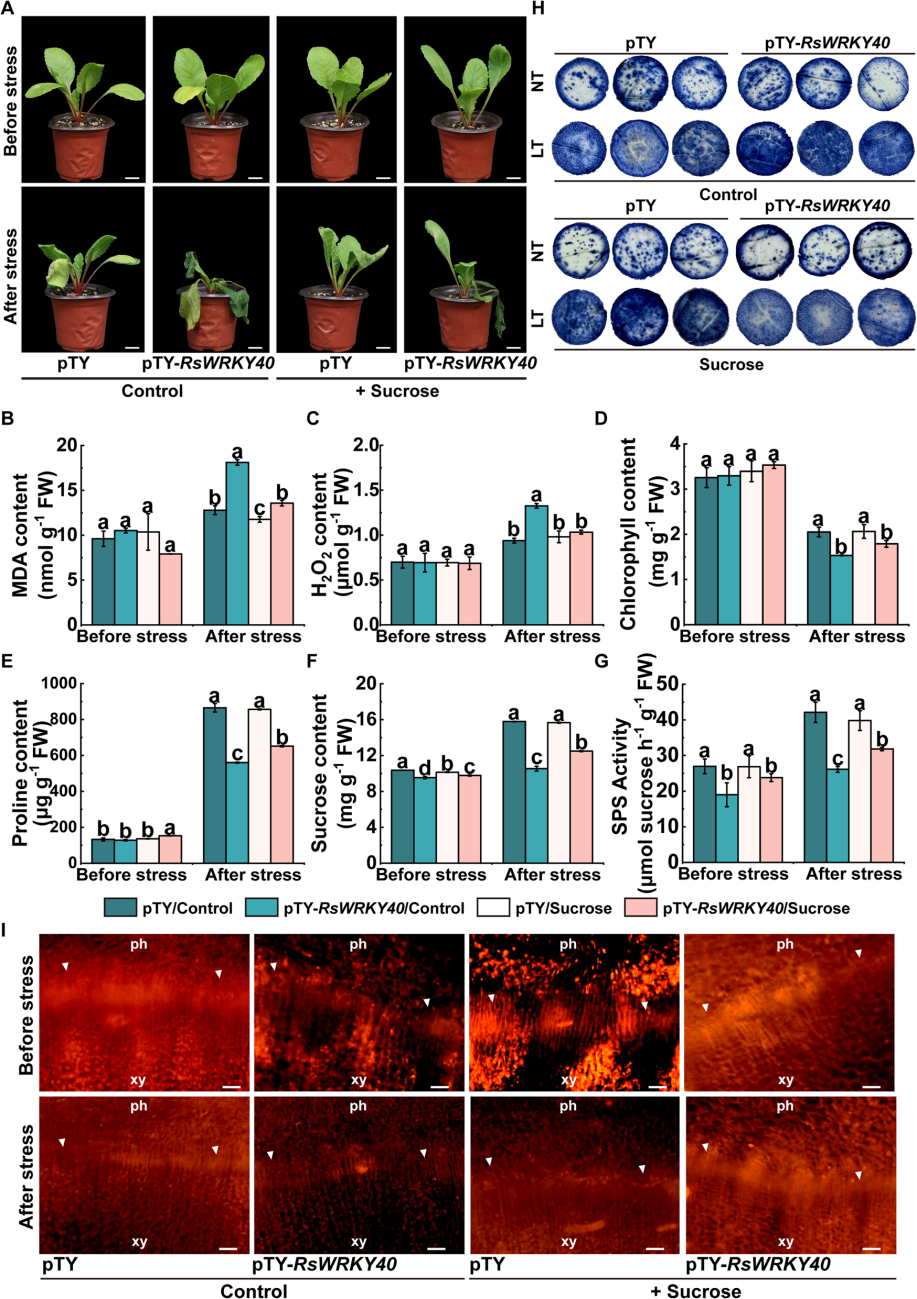
RsWRKY40 regulates cold stress response in a manner of sucrose accumulation in radish. **A** Phenotype of the *RsWRKY40*-VIGS radish and control plants, pretreatment with or without 60 mM of sucrose or mannitol (used as a control), recorded before cold stress and recovery at normal temperature for 2 d after freezing treatment. Bar: 2 cm. **B**-**H** The levels of malondialdehyde (MDA) content (**B**), H_2_O_2_ content (**C**), chlorophyll content (**D**), proline content (**E**), sucrose content (**F**), SPS activity (**G**) and in situ nitro blue tetrazolium (NBT) staining (**H**) in the *RsWRKY40*-VIGS and pTY control plants with or without sucrose supply before and after cold treatment. **I** PCNA immunolocalization in the cambium cells in the taproot of the *RsWRKY40*-silenced and control radish with or without sucrose application before and after cold stress. ph, phloem; xy, xylem; arrowhead, cambium. Bar: 200 μm. Data are presented the mean ± SD; *n* = 3

To further illustrate its function, *RsWRKY40* was knocked down by VIGS in radish. The significant downregulation of *RsWRKY40* expression and the insignificant alteration of its homolog gene expression were identified in VIGS-silenced plants, indicating the specific silencing of *RsWRKY40* in the VIGS plants (Fig. S8). Little difference was observed in the plant phenotype between the control and *RsWRKY40*-silenced radish before cold stress. However, the *RsWRKY40*-VIGS radish plants exhibited considerable leaf wilting and curling, along with a higher MDA and H_2_O_2_ content and O_2_^∙−^ accumulation and lower chlorophyll and proline concentrations compared to the pTY control under cold treatment (Fig. 7). In addition, *RsWRKY40* knockdown resulted in the downregulation of *RsSPS1* expression, which decreased the sucrose content and SPS activity (Figs. 7 and S9B). The PCNA signal was less detected in the cambium cells of the *RsWRKY40*-silenced plant taproots than in the controls after cold stress (Fig. 7I).

To investigate whether the role of *RsWRKY40* in the regulation of cold tolerance depends on *RsSPS1*-mediated sucrose synthesis, exogenous sucrose (60 mM) was supplied to the VIGS-silenced plants before cold treatment. *RsWRKY40*-silenced plants supplied with exogenous sucrose exhibited a prominently restored cold tolerance phenotype, accompanied by a decreased MDA and H_2_O_2_ content and increased chlorophyll and proline content compared with those without sucrose pretreatment (Fig. 7). The PCNA signal was elevated in the sucrose-pretreated *RsWRKY40*-silenced plants relative to the controls after cold stress (Fig. 7I). Furthermore, *RsSPS1* downregulation, sucrose content, and SPS activity were recovered in sucrose-supplied *RsWRKY40*-silenced plants compared with those without sucrose application (Figs. 7 and S9B). It can be inferred that *RsWRKY40* functions in the cold stress response and cambium activity by modulating sucrose accumulation.

### RsWRKY40 regulates cold tolerance in a CBF-dependent manner

To determine whether the CBF pathway is essential for cold stress responses in radish, bioinformatics analysis was performed and showed that *RsCBF1* and *RsCBF2* promoters contained multiple WRKY binding sites (Fig. S10A,B). Y1H showed that RsWRKY40 bound to the *RsCBF1* and *RsCBF2* promoters (Fig. 5H–J). The LUC intensity level was significantly elevated by the co-expression of the effector (*35S::RsWRKY40*) and reporter (*proRsCBF1/2-LUC*) in comparison with the controls (Fig. 5). In addition, both of *RsCBF1* and *RsCBF2* expression level were significantly down-regulated and up-regulated in the *RsWRKY40*-silenced and -overexpressed radish plants, respectively (Fig. S13). These results indicated that RsWRKY40 activates *RsCBF1* and *RsCBF2* expression by binding to their promoters.

### RsWRKY40 enhances its own transcription

**Fig. 8:**
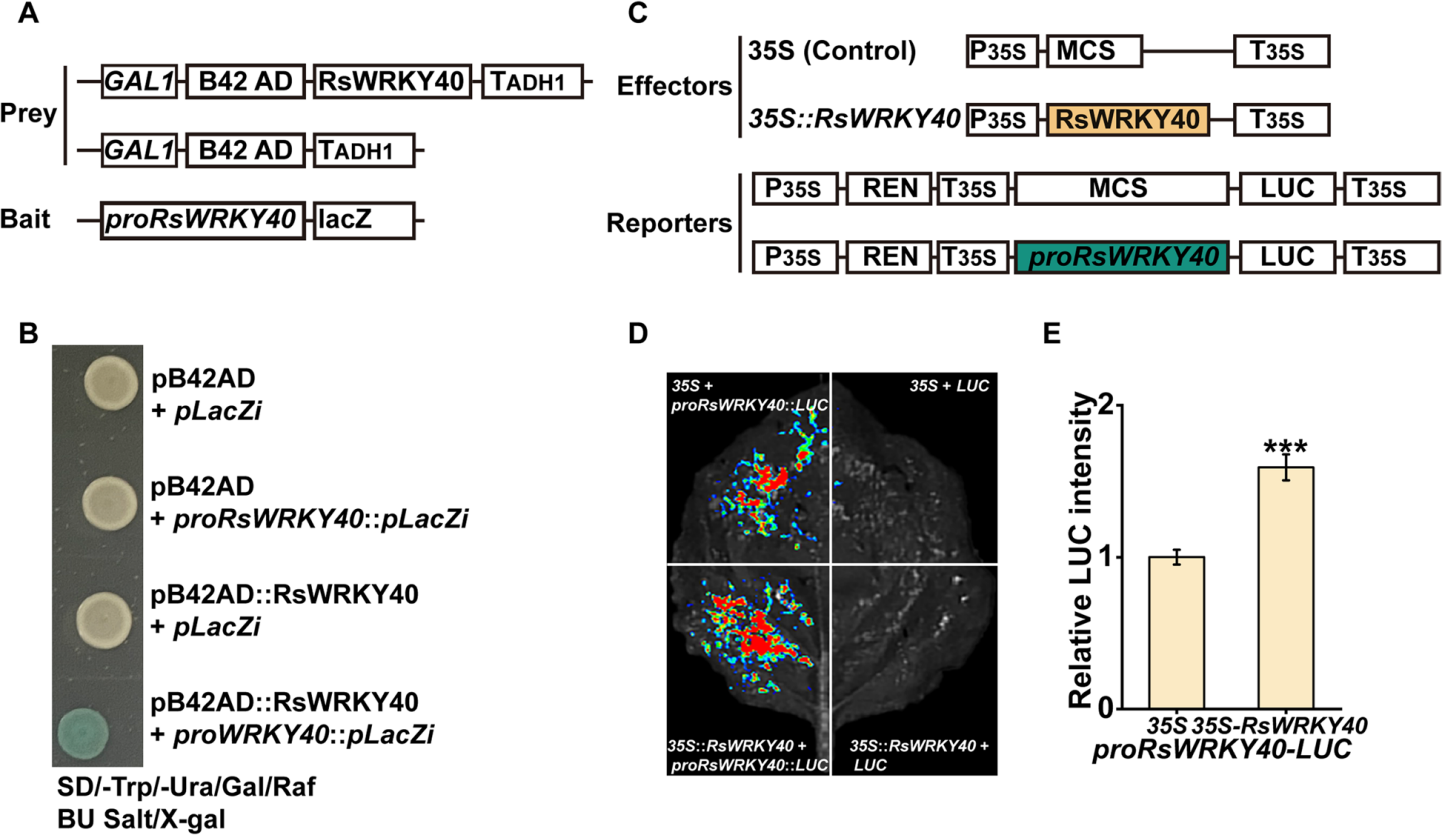
RsWRKY40 enhances its own transcription level. **A** The schematic diagram of the prey and bait constructs used in the Y1H assay. **B** RsWRKY40 binds to its own promoter. **C** The schematic diagram of the effector and reporter constructs used in the dual luciferase assay. **D**, **E** RsWRKY40 activates its own expression as indicated by LUC fluorescence images (**D**) and the relative LUC intensity (**E**). The LUC fluorescence images and relative LUC intensity were obtained by using a chemiluminescence imaging system and a Clinx IVScopeEQ Capture software, respectively. Data are presented as the mean ± SD; *n* = 3 (***P < 0.001, Student’s *t*-test)

**Fig. 9:**
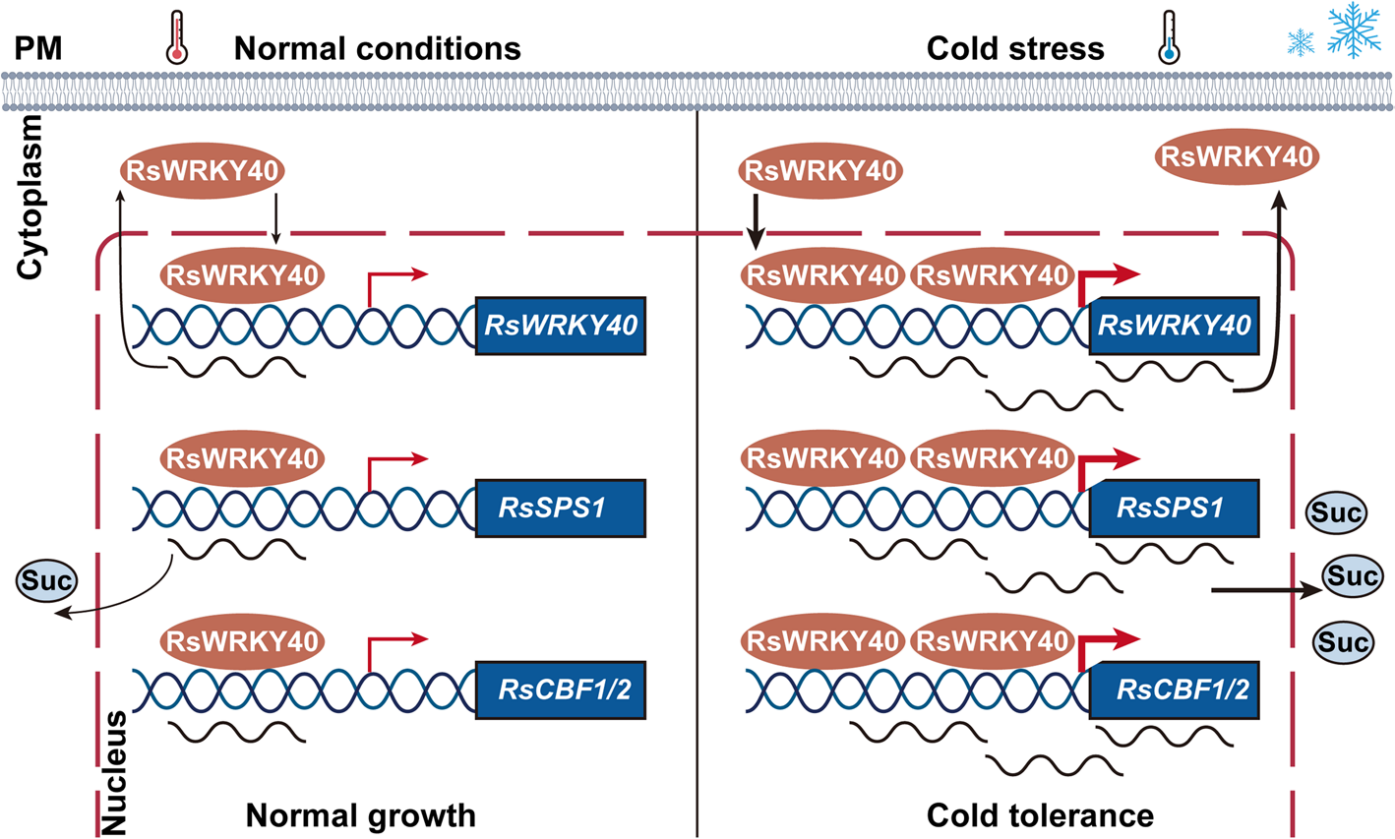
The proposed transcription model of RsWRKY40 coordinates cold stress response via *RsSPS1*-mediated sucrose accumulation and CBF-dependent pathway in radish. RsWRKY40 could regulate *RsSPS1*, *RsCBF1* and *RsCBF2* expression as well as its own transcription under normal growth conditions. RsWRKY40 is rapidly accumulated in the presence of cold stress. RsWRKY40 promotes the *RsSPS1* expression to facilitate sucrose synthesis, thereby balancing cell osmotic pressure and enhancing cold tolerance. In parallel, RsWRKY40 directly activates the expression of *RsCBF1/2* to elevate the cold tolerance in radish. RsWRKY40 also enhances its own transcription, thus forming a self-regulating loop to coordinate cold stress response

Five conserved W-box elements were detected in the *RsWRKY40* promoter sequence, suggesting that RsWRKY40 might have an auto-regulatory ability (Fig. S10C). Interestingly, the *LacZ* reporter driven by the *RsWRKY40* promoter was activated by the GAD-RsWRKY40 fusion protein, indicating that RsWRKY40 directly bound to its own promoter (Fig. 8A,B). The LUC activity of *N*. *benthamiana* leaves expressing the effector (*35S::RsWRKY40*) and reporter (*proRsWRKY40-LUC*) was 1.6-fold higher than that of the control, suggesting that RsWRKY40 activates its own expression (Fig. 8C–E) and thus producing a positive self-regulatory feedback loop to regulate the cold stress response of radish (Fig. 9).

## Discussion

SPS genes play a vital role in photosynthetic product generation, plant growth, and development. However, there is little knowledge about cold-induced transcription activation of SPS genes in cambium-driven root crops. In this study, integrating GWAS and transcriptome analysis, the role of *RsSPS1* in the modulation of sucrose synthesis and the mediation of vascular cambium activity was explored in radish. Furthermore, RsWRKY40 positively regulated *RsSPS1* expression to promote SPS activities and the sucrose content and activated *RsCBF1* and *RsCBF2* expression to coordinate the cold stress response in radish.

### *RsSPS1* is critical for cold stress response and cambium activity

Sucrose accumulation is typically affected by genetic factors, which can be dissected by genetic mapping or association studies. Through the utilization of mapping and association studies, a gene *tonoplast sugar transporter 2* (*ClTST2*) and a gene *vacuolar sugar transporter* (*CIVST1*) have been identified, whose expression level is closely related to the sugar content in watermelon fruit (Ren et al. 2017, 2020). Similarly, the *sucrose phosphate synthase* (*RsSPS1*) gene was identified using GWAS, and it was upregulated under different cold treatments (Figs. 1D and S3A). *RsSPS1* overexpression resulted in an increased SPS activity and sucrose content, while silencing it led to the opposite trend in radish, indicating that *RsSPS1* plays a vital role in the SPS activity and sucrose content in radish.

Sucrose contributes to cold tolerance by scavenging ROS and maintaining the osmotic pressure balance in plants (Bolouri-Moghaddam et al. 2010). In this study, a lower ROS content and higher sucrose content were observed in radish treated with exogenous sucrose after cold stress (Fig. 2), and *RsSPS1*-modulated sucrose synthesis contributed to cold tolerance in radish plants. Radish overexpressing *RsSPS1* had an increased sucrose and proline content and decreased ROS content after cold treatment, whereas opposite trends were observed in the silenced plants (Figs. 3 and S5). Sucrose synthesized by photosynthesis or manipulated exogenously has been shown to be a positive determinant of root growth and development and root meristem activation (Xiong et al. 2013; Lastdrager et al. 2014; Chen et al. 2016). The vascular cambium can differentiate in a periclinal direction and generate daughter cells of xylem tissue towards the organ center and phloem tissue outwards from the center (Tonn & Greb 2017). Cambium activity is critical for thickening primary and radial roots, which directly determine the biomass and yield production in most cambium-driven root crops (Jang et al. 2015). Sucrose produced in shoots functions in a shoot-to-root signal and drives lateral root development in *Arabidopsis* (Kircher & Schopfer 2023). Lateral root formation is promoted by sucrose in sunflower root cuttings cultured in sucrose solution (Kutschera & Briggs 2019). In addition, the genes related to the cell division cycle can be controlled by sucrose availability, including the upregulated expression level of cyclin genes in the G1 phase in *Arabidopsis* plants treated with exogenous sucrose (Riou-Khamlichi et al. 2000). The transcript levels of three cyclin genes are modulated by sucrose, affecting cell proliferation in the growth and development of snapdragon (*Antirrhinum majus*) (Gaudin et al. 2000). Here, radish taproot growth was promoted by exogenous sucrose treatment. The cambium activity in the taproot of exogenous sucrose-treated radish was better than that in the controls with or without cold stress (Fig. 2J). Furthermore, silencing of *RsSPS1* resulted in an obvious decrease in the PCNA signal from the cambium after cold stress (Fig. 3I). These results indicate that *RsSPS1*-modulated sucrose synthesis contributes to the maintenance of cambium activity in the radish taproot under cold stress.

### RsWRKY40 coordinates the cold stress response via CBF-independent and -dependent pathways

The sucrose biosynthesis pathway can be stimulated by low temperatures, and the elevated SPS activity results in the recovery of the photosynthesis capacity in a low-temperature environment (Seydel et al. 2022). Transgenic *Arabidopsis* plants overexpressing *CdWRKY2* or *CdSPS1* exhibit rescued photosynthetic efficiency, which regulates the cold stress response (Huang et al. 2022). In this study, *RsWRKY40* overexpression promoted the *RsSPS1* expression level, sucrose accumulation, SPS activity, and photosynthesis capacity, resulting in enhanced cold tolerance. The magnitude of changes in sucrose content and SPS activities in the *RsWRKY40*-overexpressing lines were more obvious relative to the controls under cold stress. Recently, sucrose has been shown to be critical for the balance of root meristem activity and responsible for the stimulation of cell division in plant apical meristems (Xiong & Sheen 2012; Yoon et al. 2021). Herein, the cambium activity decreased in the *RsWRKY40*-knockdown radish taproots compared to the controls after cold stress. Interestingly, the compromised *RsSPS1* expression level, sucrose content, SPS activity, and cambium activity in *RsWRKY40*-silenced radish was rescued by the exogenous sucrose supply in VIGS plants (Figs. 7 and S9B). These results indicate that the functions of RsWRKY40 in the cold stress response and the taproot cambium activity depends on the *RsSPS1*-mediated sucrose synthesis in radish.

CBFs are well-established central regulators in cold signaling, and their expression can be rapidly induced by low temperature to activate the expression of cold-regulated and downstream targeted genes, resulting in cold resilience in plants (Vogel et al. 2005; Song et al. 2021). Many TFs regulate CBF expression, including BZR1 (Li et al. 2017), CdWRKY2 (Huang et al. 2022), MdNAC104 (Mei et al. 2023), and ERF15 (Hu et al. 2024). However, the transcription regulators that directly regulate *CBF* expression are largely unreported in radish. Here, *RsCBF1* and *RsCBF2* expression was directly activated by RsWRKY40 (Fig. 5). These results indicate that RsWRKY40 positively regulates the cold stress response via the CBF-dependent pathway in radish.

### RsWRKY40-mediated transcription regulatory network of cold stress response in radish

It has been reported that WRKY TFs could bind to their own promoters to regulate their own transcription levels (Turck et al. 2004; Wu et al. 2022; Xiao et al. 2023). GhWRKY41, which is induced by the plant pathogenic fungus *Verticillium dahliae* in cotton, interacts with itself and directly activates its own transcription to regulate the cotton immune response (Xiao et al. 2023). LlWRKY22 promotes its own expression by binding to its own promoter to regulate the heat stress response in lily (Wu et al. 2022). Herein, RsWRKY40 bound to its own promoter to elevate its transcript level, thus forming a self-activating loop that positively regulated cold tolerance in radish. Cold-induced RsWRKY40 promoted *RsSPS1*-modulated sucrose accumulation to elevate cambium activity and cold tolerance. RsWRKY40 activated the expression of *RsCBF1* and *RsCBF2*, resulting in enhanced cold resilience in radish (Fig. 9). These results would provide crucial insights into the regulatory mechanism underlying sucrose accumulation and maintenance of cambium activity under cold stress, and facilitate genetic development of elite cultivars with cold tolerance in radish and other root vegetable crops.

## Materials and methods

### GWAS analysis and candidate gene identification

The population comprising 179 radish accessions was used for the association study. Genome-wide association study (GWAS) analysis was performed to detect the significant SNP associated with the soluble sugar content according to previous study (Fan et al. 2020). The mixed linear model (MLM) was used to conduct the association analysis (Lipka et al. 2012). The GWAS significance threshold was estimated to be − log_10_(*P*) = 6.0. The chromosome positions of significantly associated SNPs (− log_10_*P* > 6.0) for traits of interest were used to detect candidate genes in the radish genome (Xu et al. 2023). The pairwise linkage disequilibrium (LD) correlation was used to determine the candidate regions using the Haploview package (Barrett et al. 2004).

### Plant materials

The seeds of radish advanced inbred line ‘NAU-YH’ were germinated for 2 d at 25°C in the dark. The germinating seeds were transferred to the soil and grown in a greenhouse. In the sucrose-fed experiment, 60 mM sucrose solution was applied to 3-week-old radish plants every 2 d for 10 d.

### Stable transformation of *A. thaliana*

The *RsWRKY40* coding sequence (CDS) was inserted into the pCAMBIA1300-GFP vector to generate the RsWRKY40-GFP fusion vector. The resultant plasmid was transformed into *Agrobacterium tumefaciens* cells. Wild-type *Arabidopsis* plants were used to generate transgenic lines. The transgenic *Arabidopsis* plants were screened on Murashige and Skoog medium supplied with 36 mg/L Hygromycin B. The primer sequences are listed in Supplementary Table S1.

### Total RNA extraction and RT-qPCR

The total RNA of the collected samples was extracted using the RNA simple Total RNA Kit (TIANGEN BIOTECH, Beijing, China). The generation of first-strand complementary DNA (cDNA) was performed using the HiScript II 1st Strand cDNA Synthesis Kit (+ gDNA, wiper) (Vazyme, Nanjing, China). RT-qPCR was performed using Hieff® qPCR SYBR® Green Master Mix (Yeasen, Shanghai, China). The RT-qPCR reaction was conducted on a Roche LightCycler 480 II System (Roche, Mannheim, Germany). The 2^−ΔΔ*CT*^ method was used to calculate the relative gene expression level. *RsActin* was used as the internal reference gene. The primer sequences are listed in Supplementary Table S1.

### Promoter activity assay

The *RsWRKY40* promoter were infused upstream of the luciferase (LUC) and *β-glucuronidase* (GUS) reporter gene to generate the recombinant vectors *proRsWRKY40*-*LUC* and *proRsWRKY40*-*GUS*, respectively. The resultant plasmids were individually transformed into *Agrobacterium tumefaciens* cells (GV3101, pSoup). The bacterial cells consisting of these reconstructed vectors were infiltrated into *N. benthamiana* plants. The transformed plants were treated with or without cold stress (4℃ for 12 h), followed by LUC fluorescence analysis and GUS staining. The primer information is shown in Supplementary Table S1.

### Subcellular localization assay

The *RsWRKY40* CDS were fused into the open reading frame with green fluorescence protein (GFP). The resultant and empty vectors were individually transformed into *Agrobacterium tumefaciens*. The resuspended bacterial cells containing the recombinant constructs were transformed into tobacco plants. For subcellular observation, the infiltrated plants were subjected to 25℃ for 48 h in the dark. The fluorescence signals were obtained using a laser scanning confocal microscope (LSM900, Zeiss, Germany).

### Yeast one-hybrid (Y1H) assay

The fragments of *RsWRKY40* CDS were inserted into the pB42AD construct to generate the prey vector. To generate the bait vector, the promoters of *RsWRKY40*, *RsSPS1*, *RsCBF1*, and *RsCBF2* were infused into the pLacZi vector. The prey and bait plasmids were introduced into yeast strain EGY48 cells. These transformed yeast cells were grown and selected on SD/-Trp/-Ura medium. They were tested for *β*-galactosidase activity on a selection medium (SD/-Trp/-Ura/X-gal) (Wang et al. 2022). The primer information is shown in Table S1 in the Supporting Information.

### Dual-luciferase reporter assay

The reconstructed RsWRKY40-GFP plasmid was used as the effector. The resultant vectors of *proRsWRKY40*-*LUC*, *proRsSPS1-LUC*, *proRsCBF1*-*LUC*, and *proRsCBF2*-*LUC* were used as the reporters. The mixed *A*. *tumefaciens* suspensions harboring different recombinant vectors of effector and reporter were infiltrated into *N. benthamiana* plants to detect LUC signals (Fan et al. 2020). The images of LUC fluorescence and the relative LUC intensity were analyzed using a chemiluminescence imaging system and Clinx IVScopeEQ Capture software, respectively. The primer sequences are shown in Table S1 in Supplementary Information.

### Virus-induced gene silencing (VIGS)

To investigate the roles of *RsWRKY40* and *RsSPS1*, the VIGS system derived from turnip yellow mosaic virus (TYMV) was used to silence the genes of interest (Pflieger et al. 2008; Muntha et al. 2019). An 80-bp palindromic oligonucleotide sequence specific to the targeted genes was inserted into the *Sna*B I restriction site of pTY-S, resulting in vectors pTY-*RsWRKY40* and pTY-*RsSPS1* VIGS. The pTY empty vector and pTY-*RsWRKY40* and pTY-*RsSPS1* plasmids were infiltrated into radish plants via particle bombardment using the PDS-1000/He™ Biolistic Particle Delivery System (Bio-Rad, Hercules, CA, USA). Total genomic DNA was extracted from infiltrated radish plants. PCR amplification for the *TYMV-CP* gene (520 bp) was conducted to verify the presence of the reconstructed pTY vectors in VIGS plants. The inoculated plants with relatively lower expression levels of the targeted genes were selected and used for further analyses. In addition, 1-month-old VIGS and pTY control radish plants, with or without pretreatment with 60 mM sucrose for 12 h, were subjected to 4°C for 3 d for physiological measurement and to − 4°C for 8 h for phenotyping.

### Transient overexpression assay

Vectors pCAMBIA1300, pCAMBIA1300-RsWRKY40 and pCAMBIA1300-RsSPS1 were individually transformed into *A*. *tumefaciens* cells. The bacteria cultures containing these plasmids were resuspended in a solution buffer (200 μΜ acetosyringone, 10 mM MES, and 10 mM MgCl_2_). Two-week-old radish plants were used for the transient overexpression experiment. The bacterial solution was infiltrated into the radish cotyledons, which were incubated at 25℃ for 60 h in the dark. For the cold treatment assay, the infiltrated radish plants were subjected to 4℃ for 12 h for physiological analysis and in situ histochemical staining.

### Physiological measurement and histochemical staining

A Hydrogen Peroxide (H_2_O_2_) Content Assay Kit and the Superoxide Anion Content Assay Kit (Sangon Biotech, Shanghai, China) were used to determine the H_2_O_2_ and O_2_^∙−^ content, respectively. The malondialdehyde (MDA) content and proline content were determined according to previously reported methods (Li et al. 2020a). For histochemical staining, 3,3’-diaminobenzidine (DAB) and nitro blue tetrazolium (NBT) were used to detect the in situ accumulation of H_2_O_2_ and O_2_^∙−^content, respectively.

### Measurement of SPS activity and sucrose content

The assessment of SPS activity was carried out using previously reported methods (Zhang et al. 2019b). A 0.1 g tissue sample was ground into powder with the addition of pre-cooled extraction buffer (0.4 M ethylene glycol, 100 mM Hepes–KOH (pH 7.4), 50 mM *β*-Mercaptoethanol, 10 mM MgCl_2_, 6 mM L-Ascorbic acid, 5 mg mL^−1^ BSA, 2 mM EDTA, and 0.1% (v/v) Triton X-100). The homogenates were lightly shaken at 4℃ for 30 min, followed by centrifugation at 10,000 g for 15 min at 4℃. The supernatant was used to measure the SPS activity. An extract of 90 μL volume was added to 110 µL of the mixture (100 mM Hepes–KOH (pH 7.4), 10 mM MgCl_2_, 20 mM UDPG, 20 mM Fru-6-P, and 20 mM Glc-6-P). These reaction mixtures were incubated at 25℃ for 30 min, and 70 μL of 30% (w/v) KOH was used to terminate the reaction. The reaction tubes were incubated at 100℃ for 10 min. The SPS enzyme blanks were terminated with the addition of KOH at 0 min and incubated at 100℃ for 10 min. After cooling the reaction mixture with the addition of 1 mL of 0.2% (w/v) anthrone mix, it was incubated at 40℃ for 20 min. The absorbance of the reaction mixtures was detected at 620 nm. The sucrose produced during the reaction was used to calculate the SPS activity.

To determine the sucrose content, the collected samples were ground into powder with the addition of extraction solution. The homogenates were centrifuged, and the extraction mixtures were added to 30% (w/v) KOH and boiled for 10 min. The 0.2% (w/v) anthrone mixtures were added to the reaction mixture and incubated at 40℃ for 20 min. The absorbance of these reaction mixtures was measured at 620 nm.

### Immunolocalization assay

The visualization of cell division activity in the vascular cambium was performed using the immunolocalization assay with proliferating cell nuclear antigen (PCNA) based on previous methods (Jang et al. 2015; Dong et al. 2023).

### Statistical analysis

The data, reported as the mean ± standard deviation (SD), were analyzed using SPSS 21.0 software (IBM, Armonk, NY, USA). Differences were determined using Student’s *t*-test or one-way analysis of variance (ANOVA) based on Tukey’s multiple comparison test. Significant differences at *P* < 0.05 were indicated by different lowercase letters.

## Supplementary Information

Additional file 1: Supplementary Figure S1. The phenotype distribution of soluble sugar content showed by a histogram in the 179 radish accessions included in the GWAS population. Supplementary Figure S2. The phylogenetic relationship between RsSPS1 and AtSPS protein sequences from *A. thaliana*. The generation of phylogenetic tree was conducted by using protein sequences of RsSPS1 and AtSPS of *A. thaliana*. The MUSCLE algorithm was used for the analysis of alignment between protein sequences in MEGA 10.1.7 software. The phylogenetic tree was constructed by using the statistical methods of the neighbor-joining algorithm with 1000 bootstraps in the MEGA 10.1.7 software. The AtSPS protein sequences were obtained from the database of the Arabidopsis Information Resource (TAIR). Supplementary Figure S3. The *RsSPS1* (A) and *RsWRKY40* (B) expression level under cold stress. For gene expression analysis, 1-month-old radish plants were treated at 4°C for 0 h, 1 h, 6 h, 24 h, and 48 h in a growth chamber during a 14 h light/10 h dark. Supplementary Figure S4. The identification of the *TYMV*-CP gene and the expression level of *RsSPS1* and its homolog genes in *RsSPS1*-silenced radish. (A) The PCR amplification of the pTY-CP gene for identification of the presence of the reconstructed pTY vector in the *RsSPS1*-VIGS plants. (B) The relative expression level of *RsSPS1* analyzed by RT-qPCR in the positive pTY-*RsSPS1* transformed radish. (C) The relative expression levels of *RsSPS1* homologous genes in the *RsSPS1*-silenced plants. Supplementary Figure S5. Transient overexpression of *RsSPS1* enhances cold tolerance in radish. (A, B) The SPS activity (A) and sucrose content (B) in the radish plants transiently overexpressing *RsSPS1* (OE-*RsSPS1*) and empty vector (EV) before and after cold treatment. (C-F) The proline (C), MDA (D), H_2_O_2_ (E) and O_2_^−^ content (F) in the control and OE-*RsSPS1* lines before and after cold treatment. (G) In situ histochemical staining of nitro blue tetrazolium (NBT) (left panel) and 3,3'-diaminobenzidine (DAB) (right panel) in the control and OE-*RsSPS1* radish cotyledons with or without cold stress. Asterisks indicate the significant differences between the control and OE lines. (*P < 0.05, **P < 0.01, ***P < 0.001). Data are presented as the mean ± SD; *n* = 3. Supplementary Figure S6. The phylogenetic tree and analysis of RsWRKY40 conserved protein domain. (A) The generation of phylogenetic tree was conducted by using protein sequences of RsWRKY40 and AtWRKY from *A. thaliana*. (B) The WRKY conserved protein domain was predicted by using the RsWRKY40 protein sequence via a Web CD-Search Tool (Wang, J et al*.* 2022a). (C) The RsWRKY40 conserved domain. The WRKY conserved sequence was highlighted by bold red letters, and the zinc finger structure was shown by the black frame square and red letters. Supplementary Figure S7. The SPS activity, sucrose accumulation and cold tolerance are elevated by transient overexpression of *RsWRKY40* in radish. (A-D) The levels of MDA (A), H_2_O_2_ (B), O_2_^∙−^ (C), and proline content (D) in the radish cotyledons transiently overexpressing *RsWRKY40* (OE-*RsWRKY40*) and the control plants before and after cold treatment. (E, F) The level of sucrose content (E) and SPS activity (F) in the control and OE-*RsWRKY40* radish lines before and after cold stress. (G) In situ histochemical staining of nitro blue tetrazolium (NBT) (left panel) and 3,3'-diaminobenzidine (DAB) (right panel) in the control and OE-*RsWRKY40* plants with or without cold treatment. Asterisks indicate the significant differences between the control and OE lines. (*P < 0.05, **P < 0.01, ***P < 0.001). Data are presented as the mean ± SD; *n* = 3. Supplementary Figure S8. The identification of the *TYMV*-CP gene and the expression level of *RsWRKY40* and its homolog genes in *RsWRKY40*-silenced lines. (A) The PCR amplification of the pTY-CP gene for identification of the presence of resultant pTY plasmid in the *RsWRKY40*-VIGS plants. (B) The relative expression level of *RsWRKY40* obtained by RT-qPCR in the positive pTY-*RsWRKY40* transformed radish plants. (C) The relative expression levels of *RsWRKY40* homologous genes in the *RsWRKY40*-silenced radish plants. Supplementary Figure S9. The *RsSPS1* transcript level in the *RsWRKY40*-overexpressing (A) or -silencing (B) radish. Supplementary Figure S10. The diagram of the putative WRKY TF binding sites in the promoters of the interested genes. The W-box elements are indicated by the blue box. (A) The core sequences of WRKY TF binding sites within the *RsCBF1* promoter. W-box 1 (W1), ggGTCAAt. W-box 2 (W2), tgGTCAAt. W-box 3 (W3), gaGTCAAa. W-box 4 (W4), agGTCAAg. (B) The core sequences of WRKY TF binding sites within the *RsCBF2* promoter. W-box 1 (W1), gTTGACa. W-box 2 (W2), ggGTCAAt. (C) The core sequences of WRKY TF binding sites within the *RsWRKY40* promoter. W-box 1 (W1), caGTCAAg. W-box 2 (W2), cTTGACta. W-box 3 (W3), cTTGACtt. W-box 4 (W4), tTTGACcg. W-box 5 (W5), aaGTCAAc. (D) The core sequences of WRKY TF binding sites within the *RsSPS1* promoter. W-box 1 (W1), taGTCAAa. W-box 2 (W2), tgGTCAAa. W-box 3 (W3), taGTCAAa. W-box 4 (W4), caGTCAAa. W-box 5 (W5), caGTCAAa. Supplementary Figure. S11 Survival rate of the *RsSPS1-*silenced and control radish plants (A) *RsWRKY40*-VIGS and pTY control plants pretreated with or without sucrose (B) and *Arabidopsis* plants overexpressing *RsWRKY40* (C) recorded after cold stress. Supplementary Figure. S12 Relative staining intensity of in situ histochemical staining of nitro blue tetrazolium (NBT) and 3,3'-diaminobenzidine (DAB). (A) Relative NBT staining intensity in the control and sucrose-treated radish. (B) Relative NBT staining intensity in the control and *RsSPS1*-silenced radish plants. (C, D) Relative NBT (C) and DAB (D) staining intensity in the radish plants transiently overexpressing *RsSPS1* (OE-*RsSPS1*) and empty vector (EV). (E, F) Relative NBT (E) and DAB (F) staining intensity in the control and OE-*RsWRKY40* plants. Supplementary Figure. S13 *RsCBF1* and *RsCBF2* expression level in the *RsWRKY40*-silenced and -overexpressed radish plants.

Additional file 2: Supplementary Table S1. Primers used in this study. Supplementary Table S2. Summary of SSC content grouped by SNP genotype.

## Data Availability

The data will be available from the corresponding author upon reasonable request.

## Abbreviations

CBF: C-repeat binding factor
GFP: Green fluorescence protein
GUS: *β-glucuronidase*
GWAS: Genome-wide association study
LUC: Luciferase
PCNA: Proliferating cell nuclear antigen
ROS: Reactive oxygen species
SPS: Sucrose phosphate synthase
SSC: Soluble sugar content
VIGS: Virus-induced gene silencing
Y1H: Yeast one-hybrid
