# Supplementary figures and images for: RsWRKY40 coordinates the cold stress response by integrating RsSPS1-mediated sucrose accumulation and the CBF-dependent pathway in radish (Raphanus sativus L.)
Source: Mol Hortic. 2025 Mar 2;5:14. doi: 10.1186/s43897-024-00135-x (PMC11872316; doi:10.1186/s43897-024-00135-x)

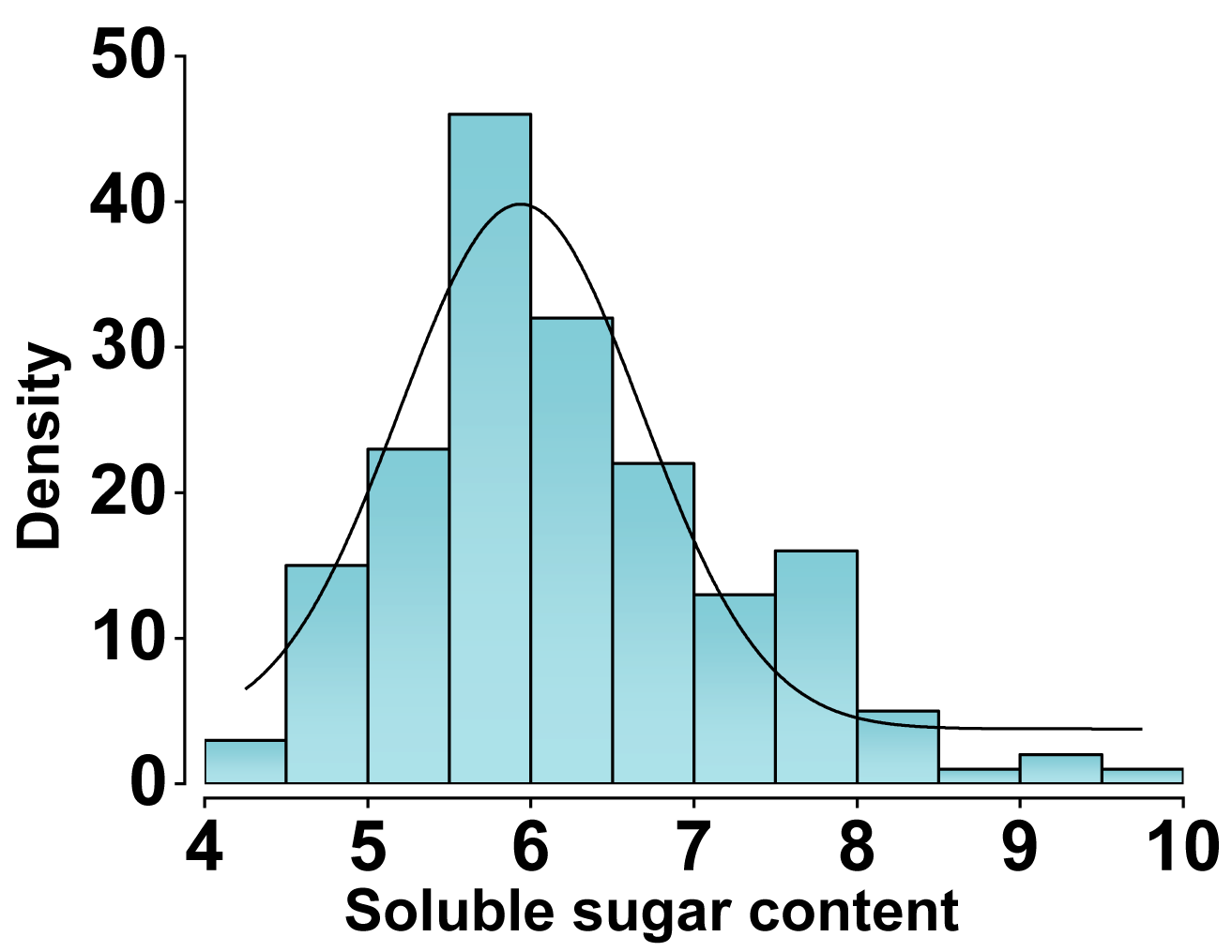

Supplement: Supplementary file 1 — Additional file 1: Supplementary Figure S1. The phenotype distribution of soluble sugar content showed by a histogram in the 179 radish accessions included in the GWAS population. Supplementary Figure S2. The phylogenetic relationship between RsSPS1 and AtSPS protein sequences from A. thaliana. The generation of phylogenetic tree was conducted by using protein sequences of RsSPS1 and AtSPS of A. thaliana. The MUSCLE algorithm was used for the analysis of alignment between protein sequences in MEGA 10.1.7 software. The phylogenetic tree was constructed by using the statistical methods of the neighbor-joining algorithm with 1000 bootstraps in the MEGA 10.1.7 software. The AtSPS protein sequences were obtained from the database of the Arabidopsis Information Resource (TAIR). Supplementary Figure S3. The RsSPS1 (A) and RsWRKY40 (B) expression level under cold stress. For gene expression analysis, 1-month-old radish plants were treated at 4°C for 0 h, 1 h, 6 h, 24 h, and 48 h in a growth chamber during a 14 h light/10 h dark. Supplementary Figure S4. The identification of the TYMV-CP gene and the expression level of RsSPS1 and its homolog genes in RsSPS1-silenced radish. (A) The PCR amplification of the pTY-CP gene for identification of the presence of the reconstructed pTY vector in the RsSPS1-VIGS plants. (B) The relative expression level of RsSPS1 analyzed by RT-qPCR in the positive pTY-RsSPS1 transformed radish. (C) The relative expression levels of RsSPS1 homologous genes in the RsSPS1-silenced plants. Supplementary Figure S5. Transient overexpression of RsSPS1 enhances cold tolerance in radish. (A, B) The SPS activity (A) and sucrose content (B) in the radish plants transiently overexpressing RsSPS1 (OE-RsSPS1) and empty vector (EV) before and after cold treatment. (C-F) The proline (C), MDA (D), H2O2 (E) and O2− content (F) in the control and OE-RsSPS1 lines before and after cold treatment. (G) In situ histochemical staining of nitro blue tetrazolium (NBT) (left p [file 43897_2024_135_MOESM1_ESM.zip › Figure-S1.tif]

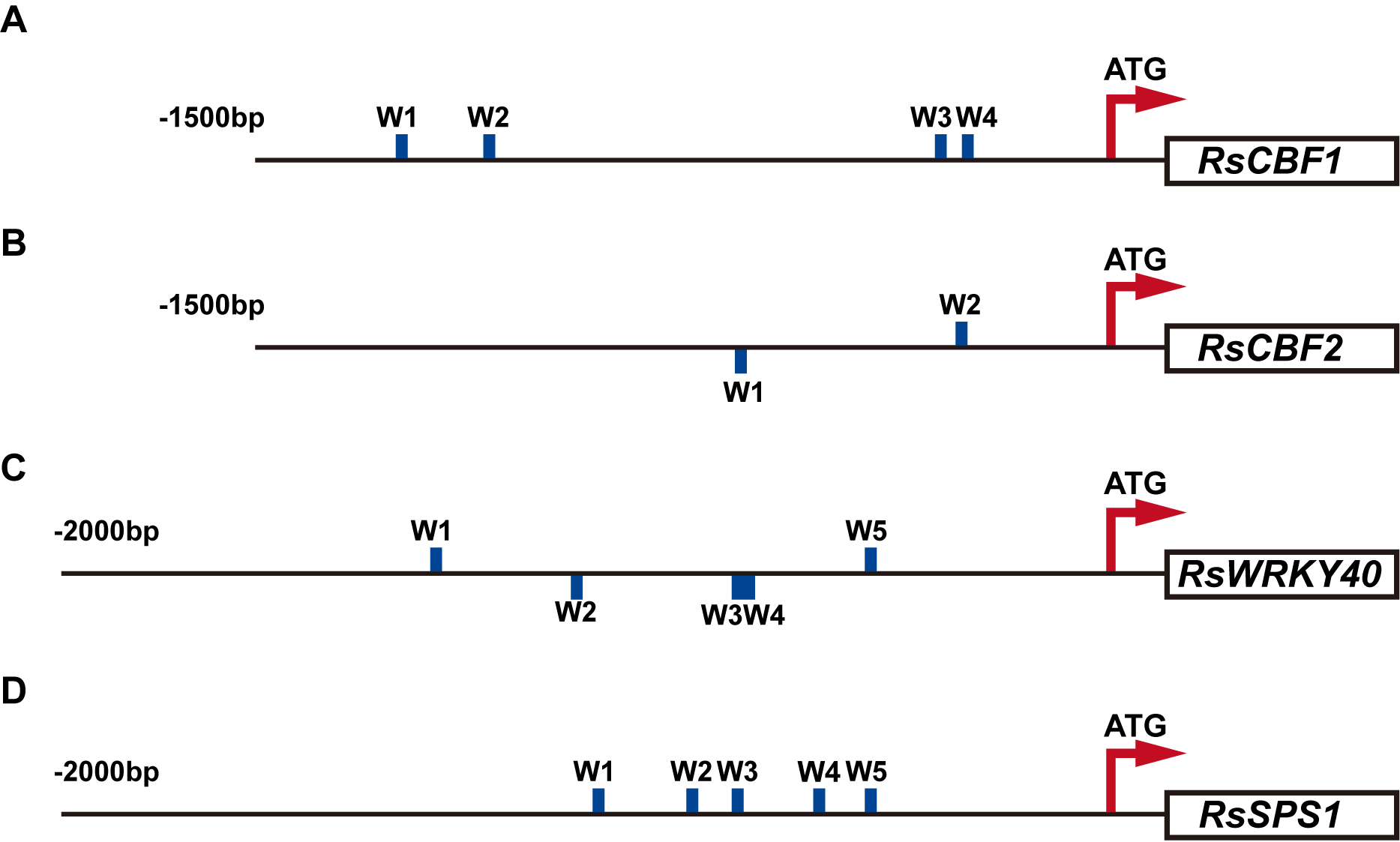

Supplement: Supplementary file 1 — Additional file 1: Supplementary Figure S1. The phenotype distribution of soluble sugar content showed by a histogram in the 179 radish accessions included in the GWAS population. Supplementary Figure S2. The phylogenetic relationship between RsSPS1 and AtSPS protein sequences from A. thaliana. The generation of phylogenetic tree was conducted by using protein sequences of RsSPS1 and AtSPS of A. thaliana. The MUSCLE algorithm was used for the analysis of alignment between protein sequences in MEGA 10.1.7 software. The phylogenetic tree was constructed by using the statistical methods of the neighbor-joining algorithm with 1000 bootstraps in the MEGA 10.1.7 software. The AtSPS protein sequences were obtained from the database of the Arabidopsis Information Resource (TAIR). Supplementary Figure S3. The RsSPS1 (A) and RsWRKY40 (B) expression level under cold stress. For gene expression analysis, 1-month-old radish plants were treated at 4°C for 0 h, 1 h, 6 h, 24 h, and 48 h in a growth chamber during a 14 h light/10 h dark. Supplementary Figure S4. The identification of the TYMV-CP gene and the expression level of RsSPS1 and its homolog genes in RsSPS1-silenced radish. (A) The PCR amplification of the pTY-CP gene for identification of the presence of the reconstructed pTY vector in the RsSPS1-VIGS plants. (B) The relative expression level of RsSPS1 analyzed by RT-qPCR in the positive pTY-RsSPS1 transformed radish. (C) The relative expression levels of RsSPS1 homologous genes in the RsSPS1-silenced plants. Supplementary Figure S5. Transient overexpression of RsSPS1 enhances cold tolerance in radish. (A, B) The SPS activity (A) and sucrose content (B) in the radish plants transiently overexpressing RsSPS1 (OE-RsSPS1) and empty vector (EV) before and after cold treatment. (C-F) The proline (C), MDA (D), H2O2 (E) and O2− content (F) in the control and OE-RsSPS1 lines before and after cold treatment. (G) In situ histochemical staining of nitro blue tetrazolium (NBT) (left p [file 43897_2024_135_MOESM1_ESM.zip › Figure-S10.tif]

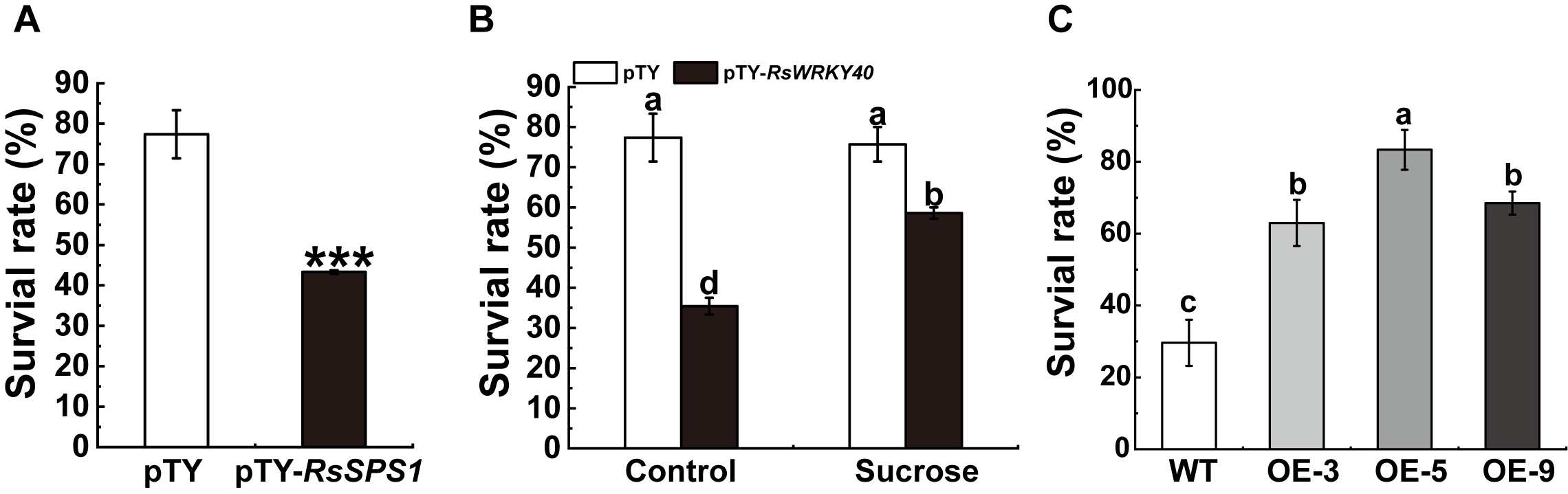

Supplement: Supplementary file 1 — Additional file 1: Supplementary Figure S1. The phenotype distribution of soluble sugar content showed by a histogram in the 179 radish accessions included in the GWAS population. Supplementary Figure S2. The phylogenetic relationship between RsSPS1 and AtSPS protein sequences from A. thaliana. The generation of phylogenetic tree was conducted by using protein sequences of RsSPS1 and AtSPS of A. thaliana. The MUSCLE algorithm was used for the analysis of alignment between protein sequences in MEGA 10.1.7 software. The phylogenetic tree was constructed by using the statistical methods of the neighbor-joining algorithm with 1000 bootstraps in the MEGA 10.1.7 software. The AtSPS protein sequences were obtained from the database of the Arabidopsis Information Resource (TAIR). Supplementary Figure S3. The RsSPS1 (A) and RsWRKY40 (B) expression level under cold stress. For gene expression analysis, 1-month-old radish plants were treated at 4°C for 0 h, 1 h, 6 h, 24 h, and 48 h in a growth chamber during a 14 h light/10 h dark. Supplementary Figure S4. The identification of the TYMV-CP gene and the expression level of RsSPS1 and its homolog genes in RsSPS1-silenced radish. (A) The PCR amplification of the pTY-CP gene for identification of the presence of the reconstructed pTY vector in the RsSPS1-VIGS plants. (B) The relative expression level of RsSPS1 analyzed by RT-qPCR in the positive pTY-RsSPS1 transformed radish. (C) The relative expression levels of RsSPS1 homologous genes in the RsSPS1-silenced plants. Supplementary Figure S5. Transient overexpression of RsSPS1 enhances cold tolerance in radish. (A, B) The SPS activity (A) and sucrose content (B) in the radish plants transiently overexpressing RsSPS1 (OE-RsSPS1) and empty vector (EV) before and after cold treatment. (C-F) The proline (C), MDA (D), H2O2 (E) and O2− content (F) in the control and OE-RsSPS1 lines before and after cold treatment. (G) In situ histochemical staining of nitro blue tetrazolium (NBT) (left p [file 43897_2024_135_MOESM1_ESM.zip › Figure-S11.tif]

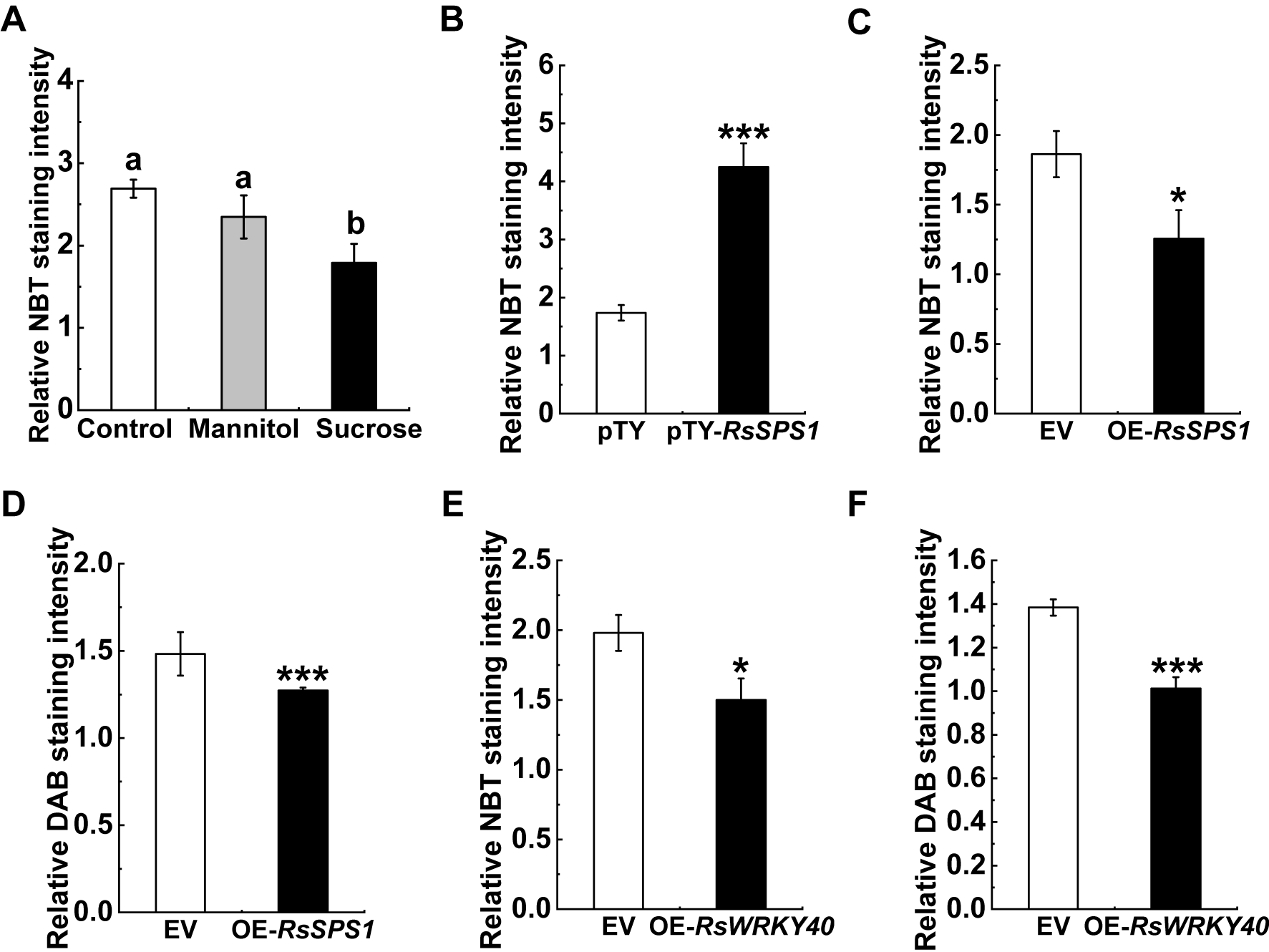

Supplement: Supplementary file 1 — Additional file 1: Supplementary Figure S1. The phenotype distribution of soluble sugar content showed by a histogram in the 179 radish accessions included in the GWAS population. Supplementary Figure S2. The phylogenetic relationship between RsSPS1 and AtSPS protein sequences from A. thaliana. The generation of phylogenetic tree was conducted by using protein sequences of RsSPS1 and AtSPS of A. thaliana. The MUSCLE algorithm was used for the analysis of alignment between protein sequences in MEGA 10.1.7 software. The phylogenetic tree was constructed by using the statistical methods of the neighbor-joining algorithm with 1000 bootstraps in the MEGA 10.1.7 software. The AtSPS protein sequences were obtained from the database of the Arabidopsis Information Resource (TAIR). Supplementary Figure S3. The RsSPS1 (A) and RsWRKY40 (B) expression level under cold stress. For gene expression analysis, 1-month-old radish plants were treated at 4°C for 0 h, 1 h, 6 h, 24 h, and 48 h in a growth chamber during a 14 h light/10 h dark. Supplementary Figure S4. The identification of the TYMV-CP gene and the expression level of RsSPS1 and its homolog genes in RsSPS1-silenced radish. (A) The PCR amplification of the pTY-CP gene for identification of the presence of the reconstructed pTY vector in the RsSPS1-VIGS plants. (B) The relative expression level of RsSPS1 analyzed by RT-qPCR in the positive pTY-RsSPS1 transformed radish. (C) The relative expression levels of RsSPS1 homologous genes in the RsSPS1-silenced plants. Supplementary Figure S5. Transient overexpression of RsSPS1 enhances cold tolerance in radish. (A, B) The SPS activity (A) and sucrose content (B) in the radish plants transiently overexpressing RsSPS1 (OE-RsSPS1) and empty vector (EV) before and after cold treatment. (C-F) The proline (C), MDA (D), H2O2 (E) and O2− content (F) in the control and OE-RsSPS1 lines before and after cold treatment. (G) In situ histochemical staining of nitro blue tetrazolium (NBT) (left p [file 43897_2024_135_MOESM1_ESM.zip › Figure-S12.tif]

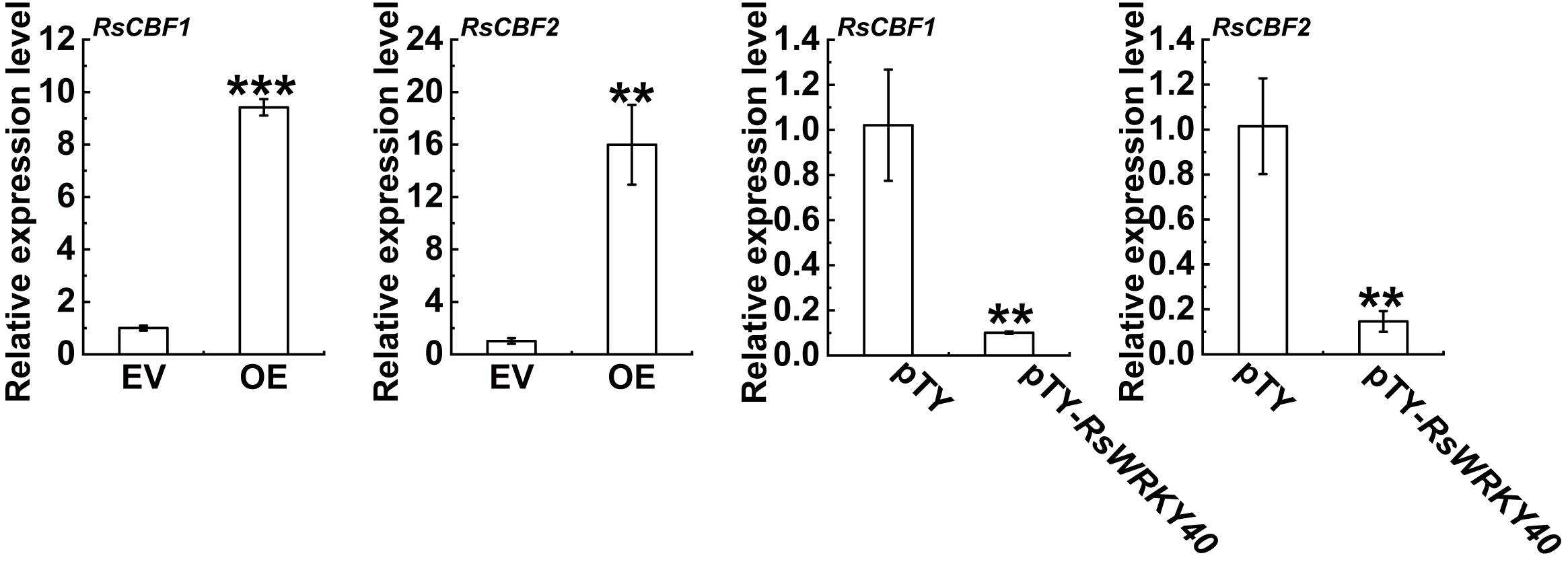

Supplement: Supplementary file 1 — Additional file 1: Supplementary Figure S1. The phenotype distribution of soluble sugar content showed by a histogram in the 179 radish accessions included in the GWAS population. Supplementary Figure S2. The phylogenetic relationship between RsSPS1 and AtSPS protein sequences from A. thaliana. The generation of phylogenetic tree was conducted by using protein sequences of RsSPS1 and AtSPS of A. thaliana. The MUSCLE algorithm was used for the analysis of alignment between protein sequences in MEGA 10.1.7 software. The phylogenetic tree was constructed by using the statistical methods of the neighbor-joining algorithm with 1000 bootstraps in the MEGA 10.1.7 software. The AtSPS protein sequences were obtained from the database of the Arabidopsis Information Resource (TAIR). Supplementary Figure S3. The RsSPS1 (A) and RsWRKY40 (B) expression level under cold stress. For gene expression analysis, 1-month-old radish plants were treated at 4°C for 0 h, 1 h, 6 h, 24 h, and 48 h in a growth chamber during a 14 h light/10 h dark. Supplementary Figure S4. The identification of the TYMV-CP gene and the expression level of RsSPS1 and its homolog genes in RsSPS1-silenced radish. (A) The PCR amplification of the pTY-CP gene for identification of the presence of the reconstructed pTY vector in the RsSPS1-VIGS plants. (B) The relative expression level of RsSPS1 analyzed by RT-qPCR in the positive pTY-RsSPS1 transformed radish. (C) The relative expression levels of RsSPS1 homologous genes in the RsSPS1-silenced plants. Supplementary Figure S5. Transient overexpression of RsSPS1 enhances cold tolerance in radish. (A, B) The SPS activity (A) and sucrose content (B) in the radish plants transiently overexpressing RsSPS1 (OE-RsSPS1) and empty vector (EV) before and after cold treatment. (C-F) The proline (C), MDA (D), H2O2 (E) and O2− content (F) in the control and OE-RsSPS1 lines before and after cold treatment. (G) In situ histochemical staining of nitro blue tetrazolium (NBT) (left p [file 43897_2024_135_MOESM1_ESM.zip › Figure-S13.tif]

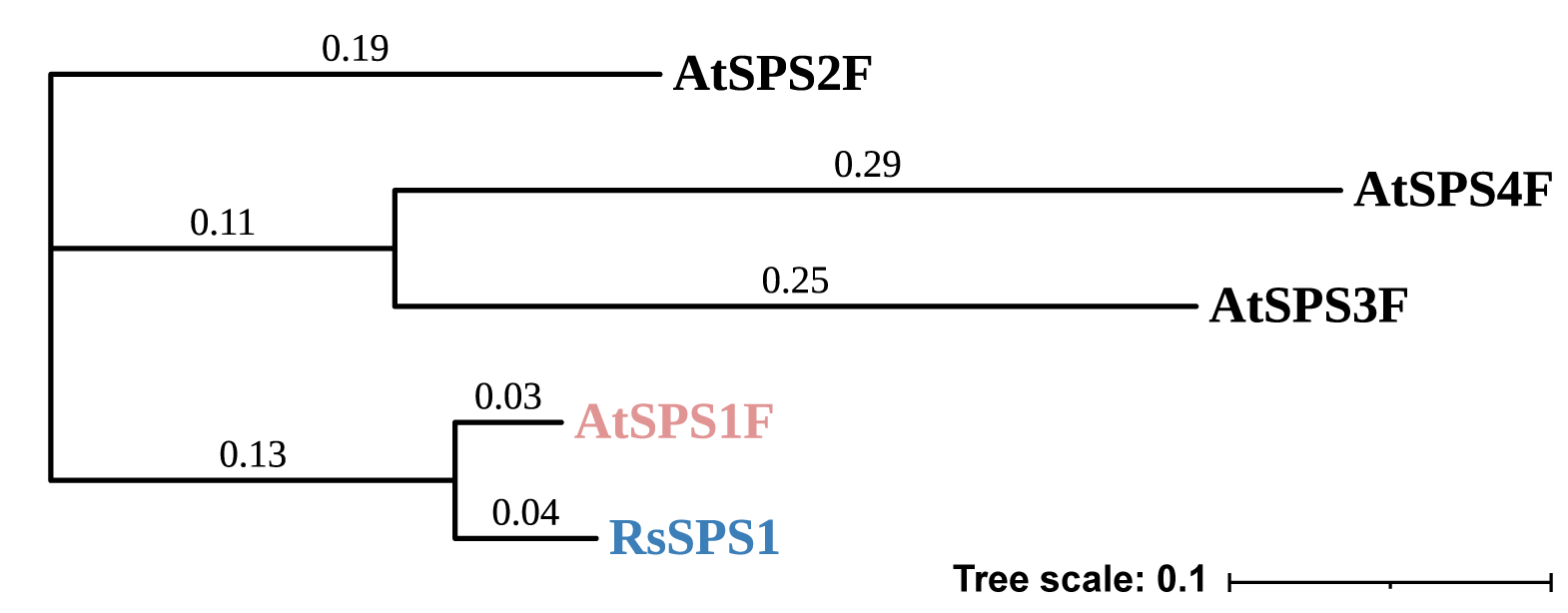

Supplement: Supplementary file 1 — Additional file 1: Supplementary Figure S1. The phenotype distribution of soluble sugar content showed by a histogram in the 179 radish accessions included in the GWAS population. Supplementary Figure S2. The phylogenetic relationship between RsSPS1 and AtSPS protein sequences from A. thaliana. The generation of phylogenetic tree was conducted by using protein sequences of RsSPS1 and AtSPS of A. thaliana. The MUSCLE algorithm was used for the analysis of alignment between protein sequences in MEGA 10.1.7 software. The phylogenetic tree was constructed by using the statistical methods of the neighbor-joining algorithm with 1000 bootstraps in the MEGA 10.1.7 software. The AtSPS protein sequences were obtained from the database of the Arabidopsis Information Resource (TAIR). Supplementary Figure S3. The RsSPS1 (A) and RsWRKY40 (B) expression level under cold stress. For gene expression analysis, 1-month-old radish plants were treated at 4°C for 0 h, 1 h, 6 h, 24 h, and 48 h in a growth chamber during a 14 h light/10 h dark. Supplementary Figure S4. The identification of the TYMV-CP gene and the expression level of RsSPS1 and its homolog genes in RsSPS1-silenced radish. (A) The PCR amplification of the pTY-CP gene for identification of the presence of the reconstructed pTY vector in the RsSPS1-VIGS plants. (B) The relative expression level of RsSPS1 analyzed by RT-qPCR in the positive pTY-RsSPS1 transformed radish. (C) The relative expression levels of RsSPS1 homologous genes in the RsSPS1-silenced plants. Supplementary Figure S5. Transient overexpression of RsSPS1 enhances cold tolerance in radish. (A, B) The SPS activity (A) and sucrose content (B) in the radish plants transiently overexpressing RsSPS1 (OE-RsSPS1) and empty vector (EV) before and after cold treatment. (C-F) The proline (C), MDA (D), H2O2 (E) and O2− content (F) in the control and OE-RsSPS1 lines before and after cold treatment. (G) In situ histochemical staining of nitro blue tetrazolium (NBT) (left p [file 43897_2024_135_MOESM1_ESM.zip › Figure-S2.tif]

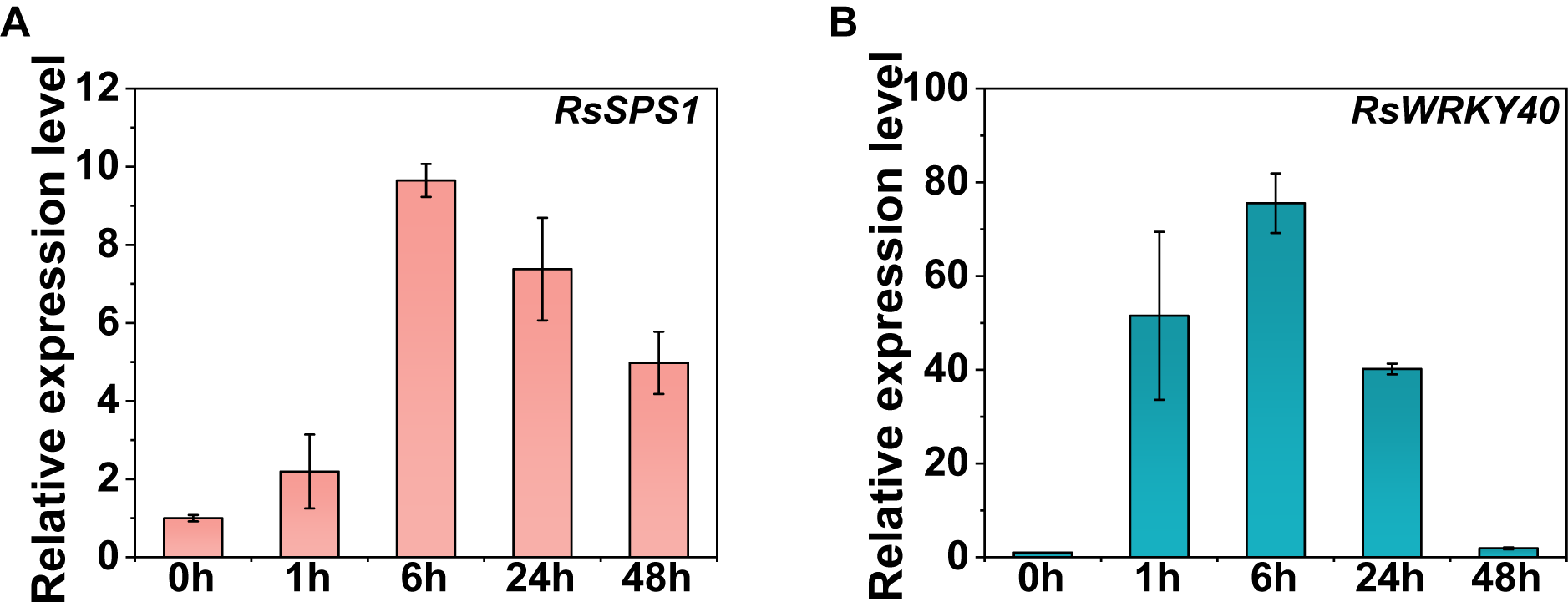

Supplement: Supplementary file 1 — Additional file 1: Supplementary Figure S1. The phenotype distribution of soluble sugar content showed by a histogram in the 179 radish accessions included in the GWAS population. Supplementary Figure S2. The phylogenetic relationship between RsSPS1 and AtSPS protein sequences from A. thaliana. The generation of phylogenetic tree was conducted by using protein sequences of RsSPS1 and AtSPS of A. thaliana. The MUSCLE algorithm was used for the analysis of alignment between protein sequences in MEGA 10.1.7 software. The phylogenetic tree was constructed by using the statistical methods of the neighbor-joining algorithm with 1000 bootstraps in the MEGA 10.1.7 software. The AtSPS protein sequences were obtained from the database of the Arabidopsis Information Resource (TAIR). Supplementary Figure S3. The RsSPS1 (A) and RsWRKY40 (B) expression level under cold stress. For gene expression analysis, 1-month-old radish plants were treated at 4°C for 0 h, 1 h, 6 h, 24 h, and 48 h in a growth chamber during a 14 h light/10 h dark. Supplementary Figure S4. The identification of the TYMV-CP gene and the expression level of RsSPS1 and its homolog genes in RsSPS1-silenced radish. (A) The PCR amplification of the pTY-CP gene for identification of the presence of the reconstructed pTY vector in the RsSPS1-VIGS plants. (B) The relative expression level of RsSPS1 analyzed by RT-qPCR in the positive pTY-RsSPS1 transformed radish. (C) The relative expression levels of RsSPS1 homologous genes in the RsSPS1-silenced plants. Supplementary Figure S5. Transient overexpression of RsSPS1 enhances cold tolerance in radish. (A, B) The SPS activity (A) and sucrose content (B) in the radish plants transiently overexpressing RsSPS1 (OE-RsSPS1) and empty vector (EV) before and after cold treatment. (C-F) The proline (C), MDA (D), H2O2 (E) and O2− content (F) in the control and OE-RsSPS1 lines before and after cold treatment. (G) In situ histochemical staining of nitro blue tetrazolium (NBT) (left p [file 43897_2024_135_MOESM1_ESM.zip › Figure-S3.tif]

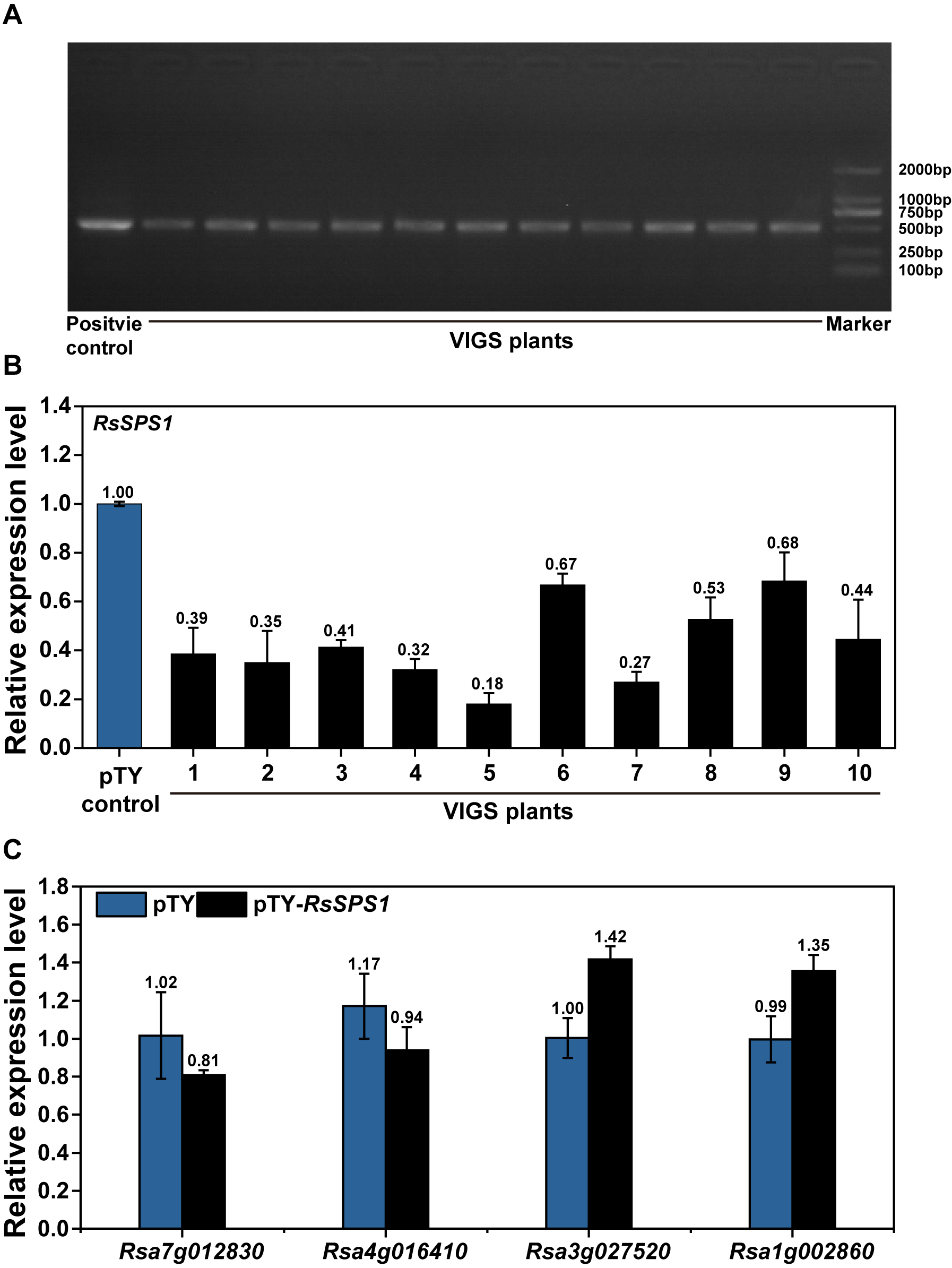

Supplement: Supplementary file 1 — Additional file 1: Supplementary Figure S1. The phenotype distribution of soluble sugar content showed by a histogram in the 179 radish accessions included in the GWAS population. Supplementary Figure S2. The phylogenetic relationship between RsSPS1 and AtSPS protein sequences from A. thaliana. The generation of phylogenetic tree was conducted by using protein sequences of RsSPS1 and AtSPS of A. thaliana. The MUSCLE algorithm was used for the analysis of alignment between protein sequences in MEGA 10.1.7 software. The phylogenetic tree was constructed by using the statistical methods of the neighbor-joining algorithm with 1000 bootstraps in the MEGA 10.1.7 software. The AtSPS protein sequences were obtained from the database of the Arabidopsis Information Resource (TAIR). Supplementary Figure S3. The RsSPS1 (A) and RsWRKY40 (B) expression level under cold stress. For gene expression analysis, 1-month-old radish plants were treated at 4°C for 0 h, 1 h, 6 h, 24 h, and 48 h in a growth chamber during a 14 h light/10 h dark. Supplementary Figure S4. The identification of the TYMV-CP gene and the expression level of RsSPS1 and its homolog genes in RsSPS1-silenced radish. (A) The PCR amplification of the pTY-CP gene for identification of the presence of the reconstructed pTY vector in the RsSPS1-VIGS plants. (B) The relative expression level of RsSPS1 analyzed by RT-qPCR in the positive pTY-RsSPS1 transformed radish. (C) The relative expression levels of RsSPS1 homologous genes in the RsSPS1-silenced plants. Supplementary Figure S5. Transient overexpression of RsSPS1 enhances cold tolerance in radish. (A, B) The SPS activity (A) and sucrose content (B) in the radish plants transiently overexpressing RsSPS1 (OE-RsSPS1) and empty vector (EV) before and after cold treatment. (C-F) The proline (C), MDA (D), H2O2 (E) and O2− content (F) in the control and OE-RsSPS1 lines before and after cold treatment. (G) In situ histochemical staining of nitro blue tetrazolium (NBT) (left p [file 43897_2024_135_MOESM1_ESM.zip › Figure-S4.tif]

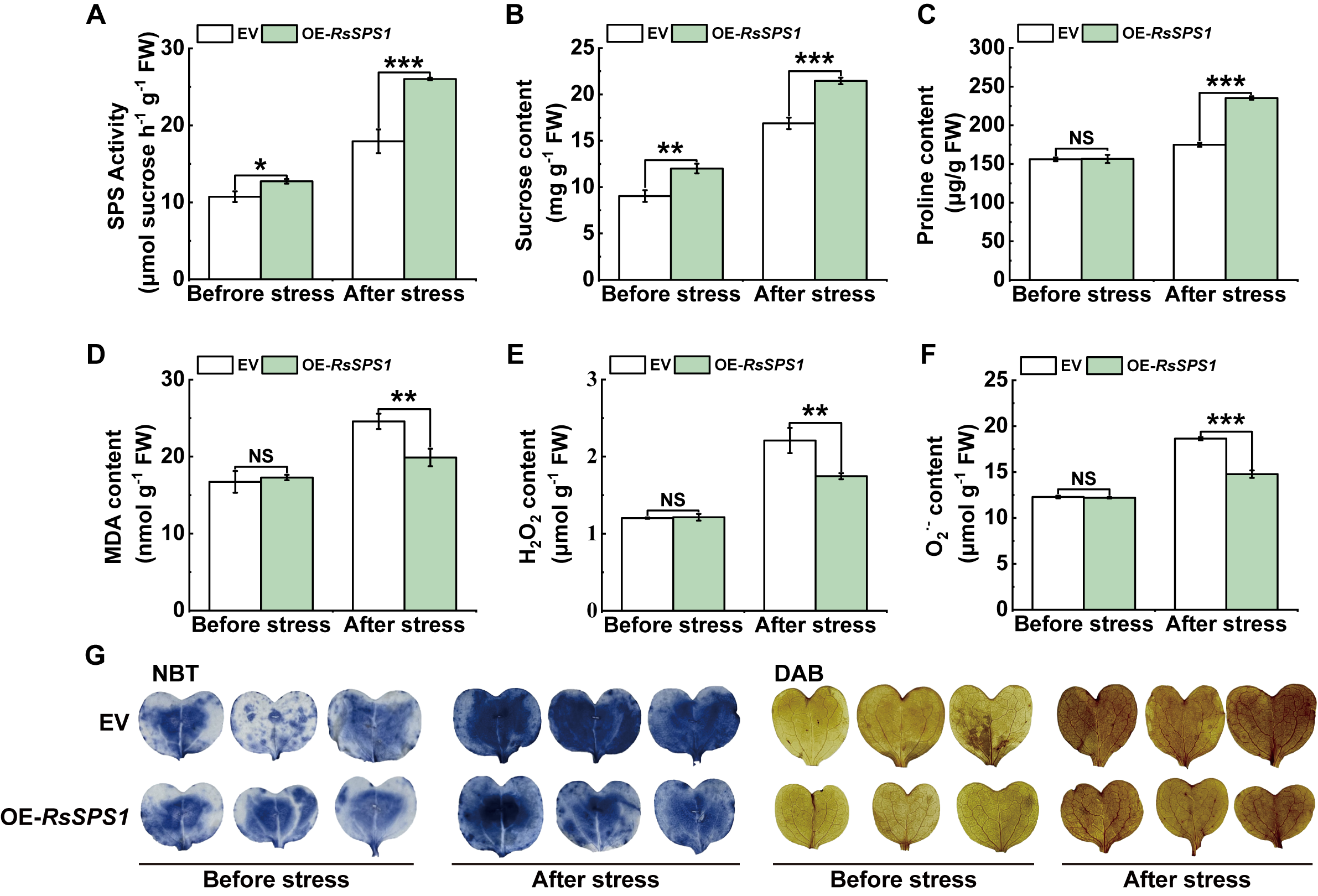

Supplement: Supplementary file 1 — Additional file 1: Supplementary Figure S1. The phenotype distribution of soluble sugar content showed by a histogram in the 179 radish accessions included in the GWAS population. Supplementary Figure S2. The phylogenetic relationship between RsSPS1 and AtSPS protein sequences from A. thaliana. The generation of phylogenetic tree was conducted by using protein sequences of RsSPS1 and AtSPS of A. thaliana. The MUSCLE algorithm was used for the analysis of alignment between protein sequences in MEGA 10.1.7 software. The phylogenetic tree was constructed by using the statistical methods of the neighbor-joining algorithm with 1000 bootstraps in the MEGA 10.1.7 software. The AtSPS protein sequences were obtained from the database of the Arabidopsis Information Resource (TAIR). Supplementary Figure S3. The RsSPS1 (A) and RsWRKY40 (B) expression level under cold stress. For gene expression analysis, 1-month-old radish plants were treated at 4°C for 0 h, 1 h, 6 h, 24 h, and 48 h in a growth chamber during a 14 h light/10 h dark. Supplementary Figure S4. The identification of the TYMV-CP gene and the expression level of RsSPS1 and its homolog genes in RsSPS1-silenced radish. (A) The PCR amplification of the pTY-CP gene for identification of the presence of the reconstructed pTY vector in the RsSPS1-VIGS plants. (B) The relative expression level of RsSPS1 analyzed by RT-qPCR in the positive pTY-RsSPS1 transformed radish. (C) The relative expression levels of RsSPS1 homologous genes in the RsSPS1-silenced plants. Supplementary Figure S5. Transient overexpression of RsSPS1 enhances cold tolerance in radish. (A, B) The SPS activity (A) and sucrose content (B) in the radish plants transiently overexpressing RsSPS1 (OE-RsSPS1) and empty vector (EV) before and after cold treatment. (C-F) The proline (C), MDA (D), H2O2 (E) and O2− content (F) in the control and OE-RsSPS1 lines before and after cold treatment. (G) In situ histochemical staining of nitro blue tetrazolium (NBT) (left p [file 43897_2024_135_MOESM1_ESM.zip › Figure-S5.tif]

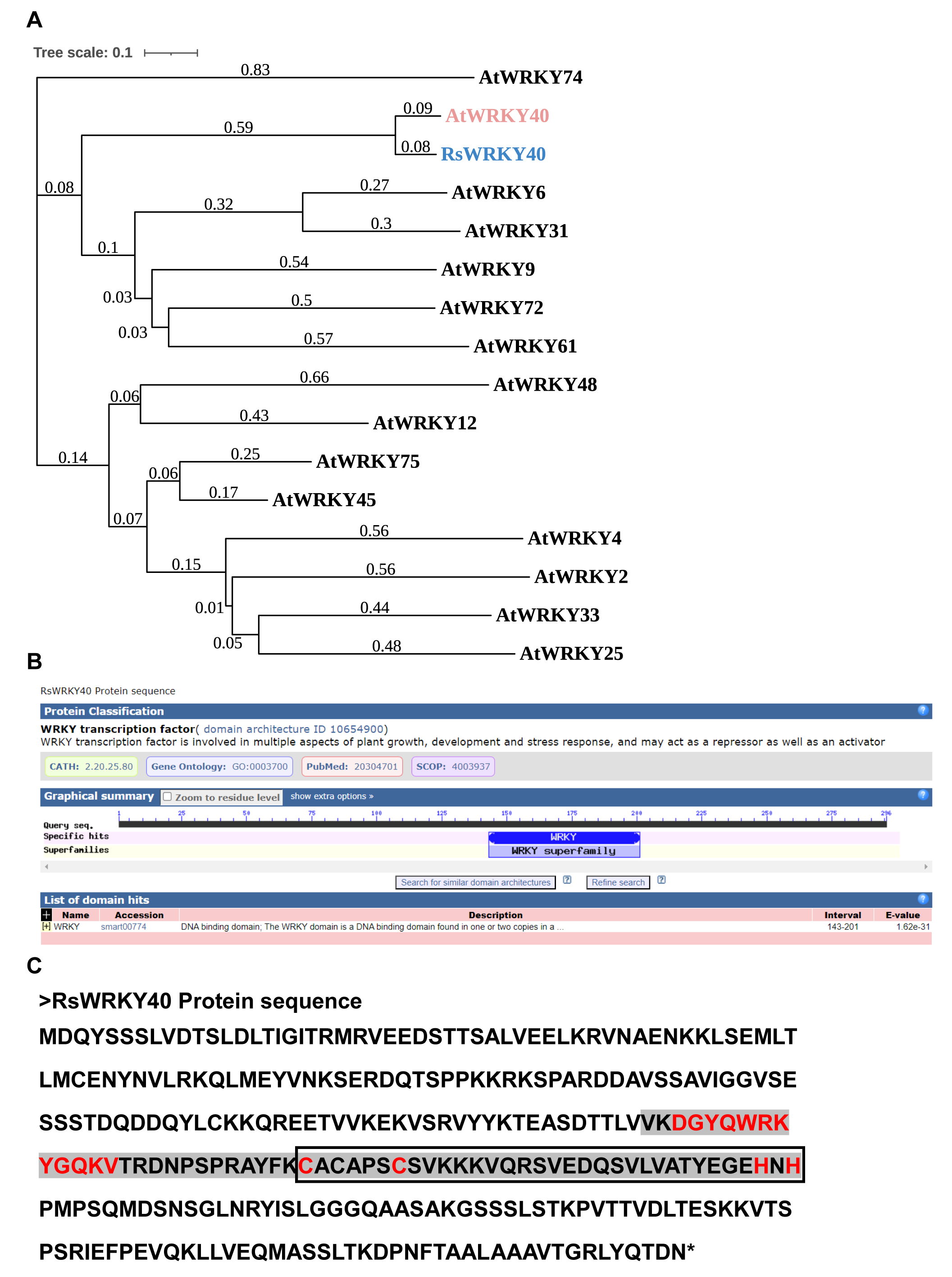

Supplement: Supplementary file 1 — Additional file 1: Supplementary Figure S1. The phenotype distribution of soluble sugar content showed by a histogram in the 179 radish accessions included in the GWAS population. Supplementary Figure S2. The phylogenetic relationship between RsSPS1 and AtSPS protein sequences from A. thaliana. The generation of phylogenetic tree was conducted by using protein sequences of RsSPS1 and AtSPS of A. thaliana. The MUSCLE algorithm was used for the analysis of alignment between protein sequences in MEGA 10.1.7 software. The phylogenetic tree was constructed by using the statistical methods of the neighbor-joining algorithm with 1000 bootstraps in the MEGA 10.1.7 software. The AtSPS protein sequences were obtained from the database of the Arabidopsis Information Resource (TAIR). Supplementary Figure S3. The RsSPS1 (A) and RsWRKY40 (B) expression level under cold stress. For gene expression analysis, 1-month-old radish plants were treated at 4°C for 0 h, 1 h, 6 h, 24 h, and 48 h in a growth chamber during a 14 h light/10 h dark. Supplementary Figure S4. The identification of the TYMV-CP gene and the expression level of RsSPS1 and its homolog genes in RsSPS1-silenced radish. (A) The PCR amplification of the pTY-CP gene for identification of the presence of the reconstructed pTY vector in the RsSPS1-VIGS plants. (B) The relative expression level of RsSPS1 analyzed by RT-qPCR in the positive pTY-RsSPS1 transformed radish. (C) The relative expression levels of RsSPS1 homologous genes in the RsSPS1-silenced plants. Supplementary Figure S5. Transient overexpression of RsSPS1 enhances cold tolerance in radish. (A, B) The SPS activity (A) and sucrose content (B) in the radish plants transiently overexpressing RsSPS1 (OE-RsSPS1) and empty vector (EV) before and after cold treatment. (C-F) The proline (C), MDA (D), H2O2 (E) and O2− content (F) in the control and OE-RsSPS1 lines before and after cold treatment. (G) In situ histochemical staining of nitro blue tetrazolium (NBT) (left p [file 43897_2024_135_MOESM1_ESM.zip › Figure-S6.tif]

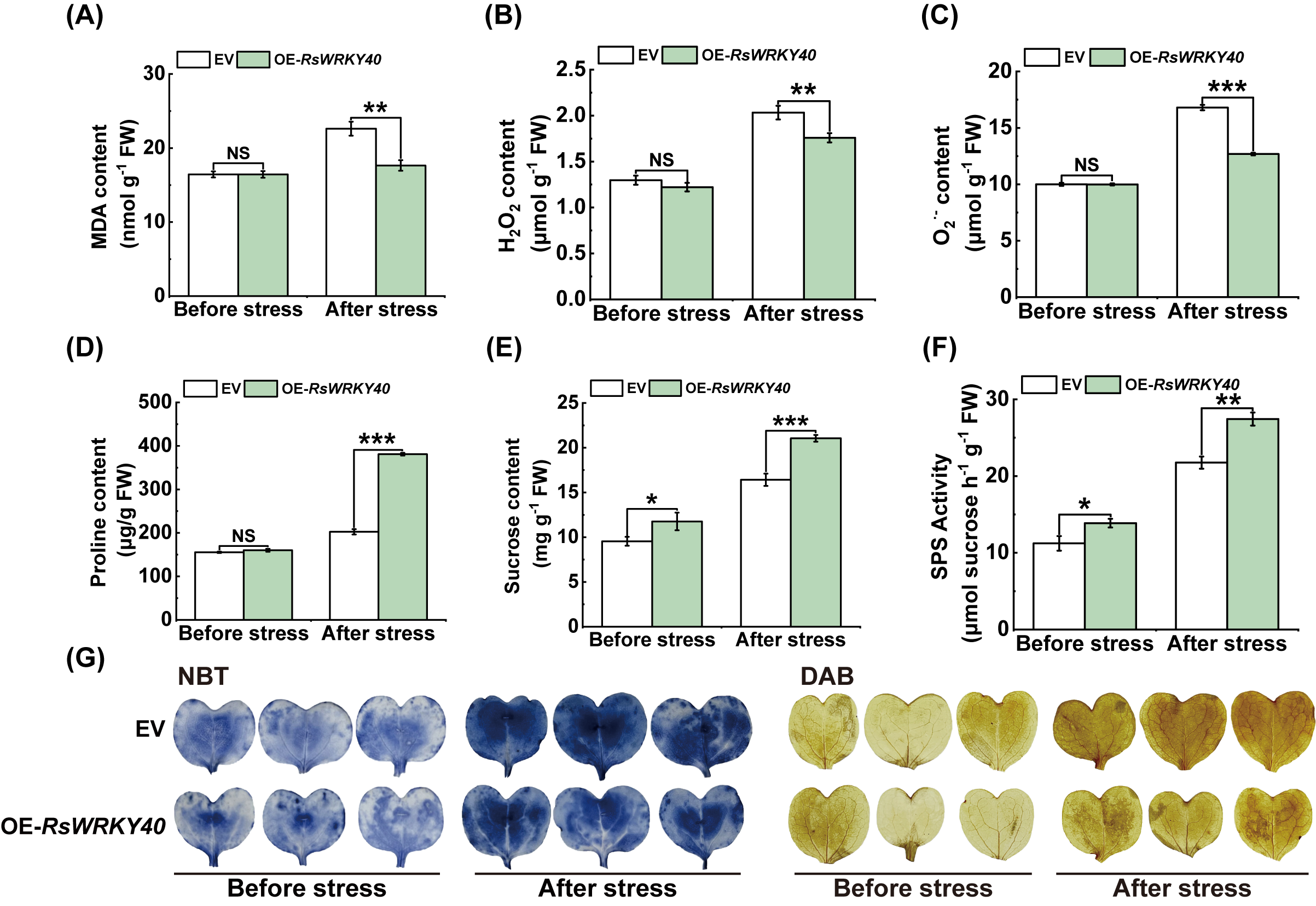

Supplement: Supplementary file 1 — Additional file 1: Supplementary Figure S1. The phenotype distribution of soluble sugar content showed by a histogram in the 179 radish accessions included in the GWAS population. Supplementary Figure S2. The phylogenetic relationship between RsSPS1 and AtSPS protein sequences from A. thaliana. The generation of phylogenetic tree was conducted by using protein sequences of RsSPS1 and AtSPS of A. thaliana. The MUSCLE algorithm was used for the analysis of alignment between protein sequences in MEGA 10.1.7 software. The phylogenetic tree was constructed by using the statistical methods of the neighbor-joining algorithm with 1000 bootstraps in the MEGA 10.1.7 software. The AtSPS protein sequences were obtained from the database of the Arabidopsis Information Resource (TAIR). Supplementary Figure S3. The RsSPS1 (A) and RsWRKY40 (B) expression level under cold stress. For gene expression analysis, 1-month-old radish plants were treated at 4°C for 0 h, 1 h, 6 h, 24 h, and 48 h in a growth chamber during a 14 h light/10 h dark. Supplementary Figure S4. The identification of the TYMV-CP gene and the expression level of RsSPS1 and its homolog genes in RsSPS1-silenced radish. (A) The PCR amplification of the pTY-CP gene for identification of the presence of the reconstructed pTY vector in the RsSPS1-VIGS plants. (B) The relative expression level of RsSPS1 analyzed by RT-qPCR in the positive pTY-RsSPS1 transformed radish. (C) The relative expression levels of RsSPS1 homologous genes in the RsSPS1-silenced plants. Supplementary Figure S5. Transient overexpression of RsSPS1 enhances cold tolerance in radish. (A, B) The SPS activity (A) and sucrose content (B) in the radish plants transiently overexpressing RsSPS1 (OE-RsSPS1) and empty vector (EV) before and after cold treatment. (C-F) The proline (C), MDA (D), H2O2 (E) and O2− content (F) in the control and OE-RsSPS1 lines before and after cold treatment. (G) In situ histochemical staining of nitro blue tetrazolium (NBT) (left p [file 43897_2024_135_MOESM1_ESM.zip › Figure-S7.tif]

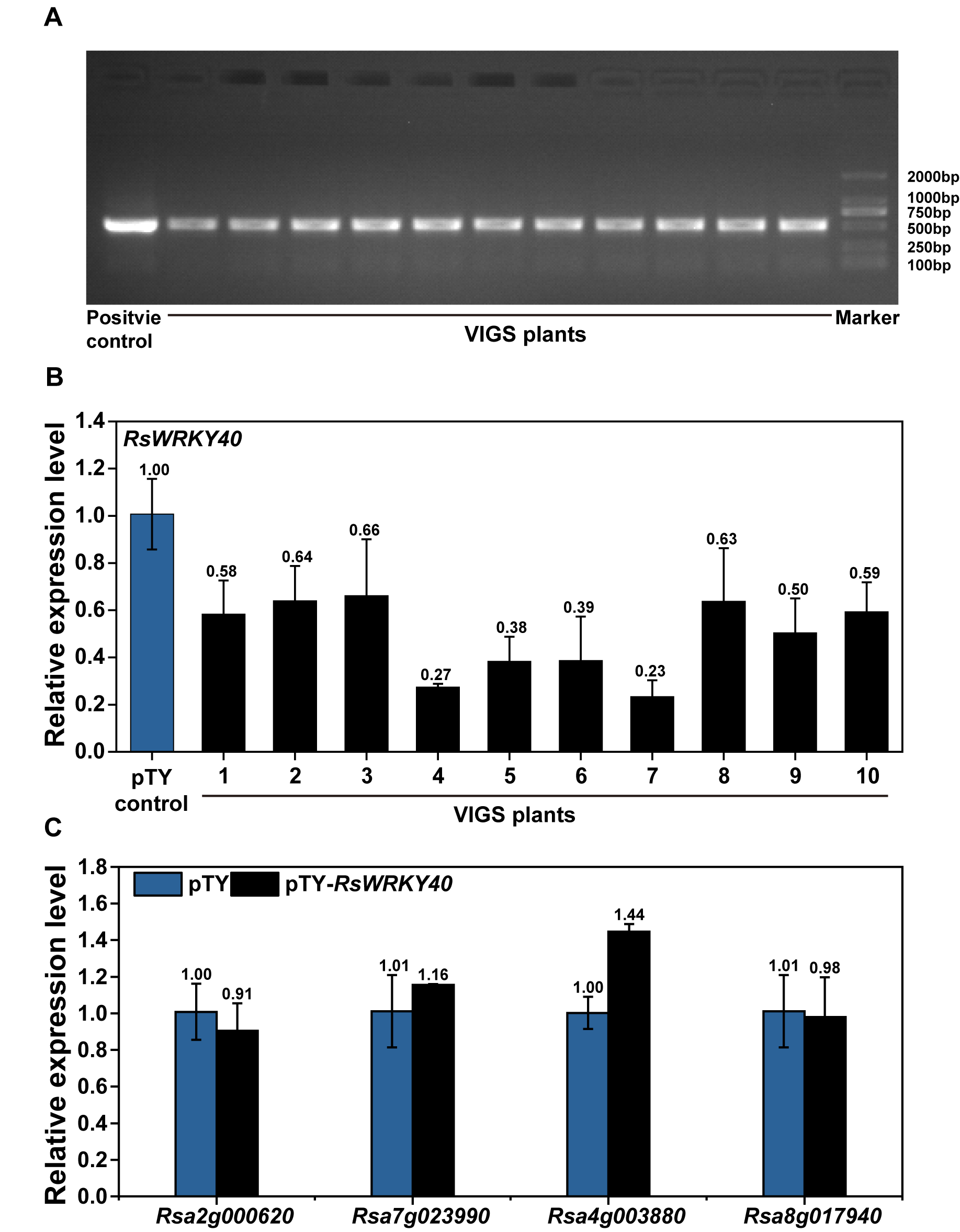

Supplement: Supplementary file 1 — Additional file 1: Supplementary Figure S1. The phenotype distribution of soluble sugar content showed by a histogram in the 179 radish accessions included in the GWAS population. Supplementary Figure S2. The phylogenetic relationship between RsSPS1 and AtSPS protein sequences from A. thaliana. The generation of phylogenetic tree was conducted by using protein sequences of RsSPS1 and AtSPS of A. thaliana. The MUSCLE algorithm was used for the analysis of alignment between protein sequences in MEGA 10.1.7 software. The phylogenetic tree was constructed by using the statistical methods of the neighbor-joining algorithm with 1000 bootstraps in the MEGA 10.1.7 software. The AtSPS protein sequences were obtained from the database of the Arabidopsis Information Resource (TAIR). Supplementary Figure S3. The RsSPS1 (A) and RsWRKY40 (B) expression level under cold stress. For gene expression analysis, 1-month-old radish plants were treated at 4°C for 0 h, 1 h, 6 h, 24 h, and 48 h in a growth chamber during a 14 h light/10 h dark. Supplementary Figure S4. The identification of the TYMV-CP gene and the expression level of RsSPS1 and its homolog genes in RsSPS1-silenced radish. (A) The PCR amplification of the pTY-CP gene for identification of the presence of the reconstructed pTY vector in the RsSPS1-VIGS plants. (B) The relative expression level of RsSPS1 analyzed by RT-qPCR in the positive pTY-RsSPS1 transformed radish. (C) The relative expression levels of RsSPS1 homologous genes in the RsSPS1-silenced plants. Supplementary Figure S5. Transient overexpression of RsSPS1 enhances cold tolerance in radish. (A, B) The SPS activity (A) and sucrose content (B) in the radish plants transiently overexpressing RsSPS1 (OE-RsSPS1) and empty vector (EV) before and after cold treatment. (C-F) The proline (C), MDA (D), H2O2 (E) and O2− content (F) in the control and OE-RsSPS1 lines before and after cold treatment. (G) In situ histochemical staining of nitro blue tetrazolium (NBT) (left p [file 43897_2024_135_MOESM1_ESM.zip › Figure-S8.tif]

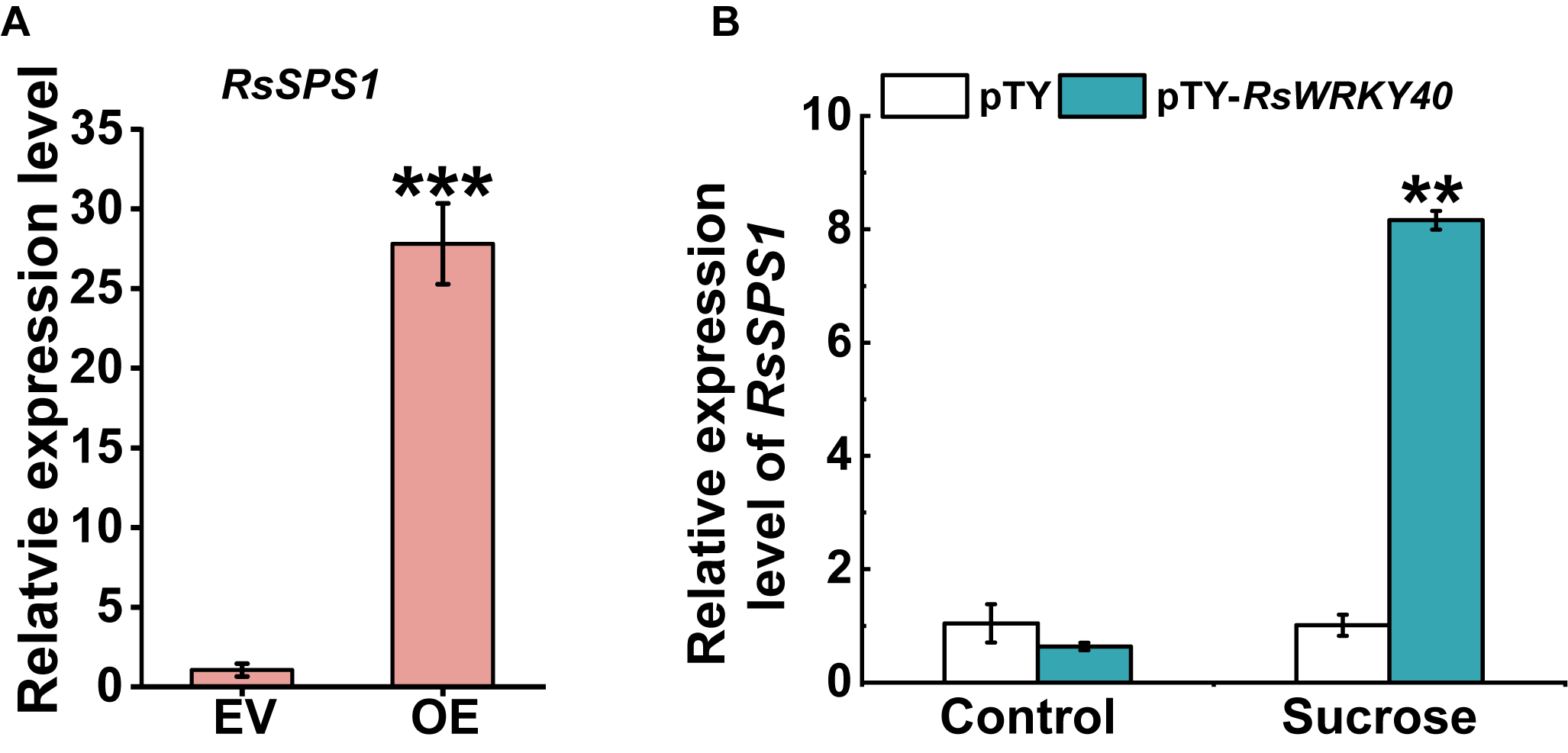

Supplement: Supplementary file 1 — Additional file 1: Supplementary Figure S1. The phenotype distribution of soluble sugar content showed by a histogram in the 179 radish accessions included in the GWAS population. Supplementary Figure S2. The phylogenetic relationship between RsSPS1 and AtSPS protein sequences from A. thaliana. The generation of phylogenetic tree was conducted by using protein sequences of RsSPS1 and AtSPS of A. thaliana. The MUSCLE algorithm was used for the analysis of alignment between protein sequences in MEGA 10.1.7 software. The phylogenetic tree was constructed by using the statistical methods of the neighbor-joining algorithm with 1000 bootstraps in the MEGA 10.1.7 software. The AtSPS protein sequences were obtained from the database of the Arabidopsis Information Resource (TAIR). Supplementary Figure S3. The RsSPS1 (A) and RsWRKY40 (B) expression level under cold stress. For gene expression analysis, 1-month-old radish plants were treated at 4°C for 0 h, 1 h, 6 h, 24 h, and 48 h in a growth chamber during a 14 h light/10 h dark. Supplementary Figure S4. The identification of the TYMV-CP gene and the expression level of RsSPS1 and its homolog genes in RsSPS1-silenced radish. (A) The PCR amplification of the pTY-CP gene for identification of the presence of the reconstructed pTY vector in the RsSPS1-VIGS plants. (B) The relative expression level of RsSPS1 analyzed by RT-qPCR in the positive pTY-RsSPS1 transformed radish. (C) The relative expression levels of RsSPS1 homologous genes in the RsSPS1-silenced plants. Supplementary Figure S5. Transient overexpression of RsSPS1 enhances cold tolerance in radish. (A, B) The SPS activity (A) and sucrose content (B) in the radish plants transiently overexpressing RsSPS1 (OE-RsSPS1) and empty vector (EV) before and after cold treatment. (C-F) The proline (C), MDA (D), H2O2 (E) and O2− content (F) in the control and OE-RsSPS1 lines before and after cold treatment. (G) In situ histochemical staining of nitro blue tetrazolium (NBT) (left p [file 43897_2024_135_MOESM1_ESM.zip › Figure-S9.tif]
